# Submaximal Fitness Test in Team Sports: A Systematic Review and Meta-Analysis of Exercise Heart Rate Measurement Properties

**DOI:** 10.1186/s40798-023-00564-w

**Published:** 2023-03-24

**Authors:** Tzlil Shushan, Ric Lovell, Martin Buchheit, Tannath J. Scott, Steve Barrett, Dean Norris, Shaun J. McLaren

**Affiliations:** 1 School of Health Sciences, Western Sydney University, Sydney, NSW Australia; 2 Faculty of Science, Medicine and Health, University of Wollongong, Wollongong, NSW Australia; 3 HIIT Science, Revelstoke, BC Canada; 4 Laboratory of Sport, Expertise and Performance, French National Institute of Sport (INSEP), 7370 Paris, France; 5 Kitman Labs, Performance Research Intelligence Initiative, Dublin, Ireland; 6 Institute for Health and Sport, Victoria University, Melbourne, VIC Australia; 7 Netball Australia, Victoria, Australia; 8 Carnegie Applied Rugby Research (CARR) Centre, Institute for Sport, Physical Activity and Leisure, Leeds Beckett University, Leeds, UK; 9 Department of Sport Science Innovation, Playermaker, London, UK; 10 Newcastle Falcons Rugby Club, Newcastle Upon Tyne, UK; 11 Institute of Sport, Manchester Metropolitan University, Manchester, UK

**Keywords:** Submaximal fitness tests, Exercise heart rate, Meta-analysis, Measurement properties

## Abstract

**Background:**

Submaximal fitness tests (SMFT) are a pragmatic approach for evaluating athlete’s physiological state, due to their time-efficient nature, low physiological burden and relative ease of administration in team sports settings. While a variety of outcome measures can be collected during SMFT, exercise heart rate (HRex) is the most popular. Understanding the measurement properties of HRex can support the interpretation of data and assist in decision making regarding athlete’s current physiological state and training effects.

**Objectives:**

The aims of our systematic review and meta-analysis were to: (1) establish meta-analytic estimates of SMFT HRex reliability and convergent validity and (2) examine the moderating influence of athlete and protocol characteristics on the magnitude of these measurement properties.

**Methods:**

We conducted a systematic literature search with MEDLINE, Scopus and Web of Science databases for studies published up until January 2022 since records began. Studies were considered for inclusion when they  included team sports athletes and the reliability and/or convergent validity of SMFT HRex was investigated. Reliability statistics included the group mean difference (MD), typical error of measurement (TE) and intraclass correlation coefficient (ICC) derived from test–retest(s) designs. Pearson’s correlation coefficient (*r*) describing the relationship between SMFT HRex and a criterion measure of endurance performance was used as the statistic for convergent validity. Qualitative assessment was conducted using risk of bias assessment tool for non-randomised studies. Mixed-effects, multilevel hierarchical models combined with robust variance estimate tests were performed to obtain pooled measurement property estimates, effect heterogeneity, and meta-regression of modifying effects.

**Results:**

The electronic search yielded 21 reliability (29 samples) and 20 convergent validity (29 samples) studies that met the inclusion criteria. Reliability meta-analysis indicated good absolute (MD = 0.5 [95% CI 0.1 to 0.9] and TE = 1.6 [95% CI 1.4 to 1.9] % points), and high relative (ICC = 0.88 [95% CI 0.84 to 0.91]) reliability. Convergent validity meta-analysis indicated an inverse, large relationship (*r* = − 0.58 [95% CI − 0.62 to − 0.54]) between SMFT HRex and endurance tests performance. Meta-regression analyses suggested no meaningful influence of SMFT protocol or athlete characteristics on reliability or convergent validity estimates.

**Conclusions:**

Submaximal fitness test HRex is a reliable and valid proxy indicator of endurance performance in team sport athletes. Athlete and SMFT protocol characteristics do not appear to have a meaningful effect on these measurement properties. Practitioners may implement SMFT HRex for monitoring athlete’s physiological state by using our applied implications to guide the interpretation of data in practice. Future research should examine the utility of SMFT HRex to track within-athlete changes in aerobic capacity, as well as any further possible effects of SMFT protocols design elements or HRex analytical methods on measurement properties.

*Registration* Protocol registration can be found in Open Science Framework and available through https://doi.org/10.17605/OSF.IO/9C2JV.

**Supplementary Information:**

The online version contains supplementary material available at 10.1186/s40798-023-00564-w.

## Key Points

- Exercise heart rate (HRex) during submaximal fitness tests is a reliable and valid proxy measure of endurance performance in team sports.
- Athlete and test protocol-related characteristics do not appear to meaningfully affect HRex measurement properties.
- Our findings provide implications for test protocol selection, as well as conceptual and statistical data interpretations when using HRex for evaluating athlete’s physiological state in team sports.

## Introduction

Quantifying athlete’s responses to training programmes is an integral part of the training process of team sports [1]. Assessing aerobic-oriented training effects is preferably made via maximal exhaustive tests administered in field-based or laboratory conditions, with higher physiological or performance outcomes indicative of improved aerobic capacity or adaptation to a training programme [2, 3]. However, given the limited viability of repeated maximal endurance performance tests in team sports, there is a growing interest in collecting proxy (surrogate) outcome measures of physiological capacities during alternative, time-efficient and non-exhaustive exercise assessments [4, 5].

Submaximal Fitness Tests (SMFT) provide a pragmatic approach for evaluating physiological state by assessing an athlete’s internal load or responses to a standardised physical stimulus [6, 7]. We recently undertook a review of the literature on SMFT in team sports [8] and identified many variations in protocols, outcome measures and monitoring purposes. Exercise heart rate (HRex) is the most utilised SMFT outcome measure, perhaps due to the strong relationship to oxygen uptake during a continuous, (intended steady-state) exercise [4], supporting its use as a surrogate marker of aerobic capacity of cardiovascular fitness [4, 9]. Although various resting, exercise and recovery HR-derived measures may provide an insight into the cardiac autonomic nervous system state [10], HRex has also been proposed as a marker of shorter-term physiological stress given its sensitivity to factors such as an exposure to extreme environments (e.g., heat, altitude) [11, 12] or a training-induced overreaching state [10, 13]. Therefore, SMFT HRex might provide practitioners with a valuable insight into athlete monitoring in team sports [4, 9].

Understanding the measurement properties of an outcome measure is a fundamental aspect in sports performance testing and monitoring [14]. Reliability refers to the consistency and reproducibility of an outcome measure [15, 16]. This is typically assessed using test–retest measurements [17] and quantifies the expected ‘noise’ (biological or measurement error) of the outcome measure [15, 17], which can subsequently assist in establishing meaningful changes for decision making [4]. Validity refers to the extent to which a measure represents the variable or construct it is intended to and includes several dimensions. For example, construct validity is the extent to which variables are reflective of theoretical constructs described by constitutive definitions and operationalizations. A sub-domain of construct validity is convergent validity—the degree to which two measures of a theoretical construct that should be related, are in fact related [18]. Within the SMFT framework, HRex should, theoretically at least, be associated with both positive [6] and negative [19] training effects.

To better understand the utility of SMFT HRex as a monitoring tool, a thorough knowledge of its measurement properties is required. There have been a considerable number of studies examining the reliability and convergent validity of SMFT HRex in team sport athletes; however, the overall (pooled) estimates of these measurement properties, the associated between- and within-study variance, and the influence of modifying effects have yet to be established. Therefore, the aims of our current paper are: (1) to provide the first meta-analysis of SMFT HRex reliability (absolute and relative) and convergent validity; and (2) to examine the influence of athlete’s and SMFT characteristics on these measurement properties.

## Methods

### Registration and Search Strategy

Our systematic review and meta-analysis was conducted in accordance with PRISMA (Preferred Reporting Items for Systematic reviews and Meta-Analyses) 2020 guidelines [20] (PRISMA checklist items are available in the Additional file 1), and was prospectively registered in Open Science Framework (available through https://doi.org/10.17605/OSF.IO/9C2JV). Details on the registration process and changes made between the original to final protocol are available in Additional file 2. The research question for this review was developed as part of an iterative process originated in our previous review [8]. For this, we updated the systematic searches with an extension of the inclusion and exclusion criteria (Table 1). The electronic databases MEDLINE, Scopus and Web of Science were searched on multiple occasions, commencing in January 2020 and finalised in January 2022, denoting the earliest search date of the original review and the most updated searches for the meta-analysis, respectively. Detailed descriptions presenting the search strategy and results are provided in the Additional file 2.

**Table 1:** Meta-analysis inclusion and exclusion criteria

Criteria	Inclusion	Description or reasons for exclusion
1	Original research published in peer-reviewed journal	Excluded non-investigation studies such as reviews, book chapters, abstracts, theses, opinion piece, surveys, letters to editor, etc.
2	Studies published until January 2022	In accordance with our most updated searches, studies published from the inception up until January 2022 were considered for inclusion, including studies published in the *Epub ahead of print* format
3	Available in English language	Full text is in English
4	Population	Team sport athletes with no restriction of age, level and sex
5	Study design	*Test–retest designs*
		Studies examining the reliability of SMFT HRex in controlled settings (e.g., scheduling, training context) and reporting absolute (group mean difference [MD], typical error of measurement [TE]), relative (intraclass correlation coefficient [ICC]) effect estimates, or both
		*Correlational designs*
		Studies examining the convergent validity of SMFT HRex with reference to a maximal endurance performance test and reporting correlational statistic (correlation coefficient [r]) of this relationship
6	Settings	SMFT were administered either in laboratory, indoor/outdoor field-based formats or combination of two
7	SMFT	Any SMFT including cycling, running, or standardised drills and games (refer to Fig. 2, ‘SMFT protocol categories’)
	*Intensity*	Excluded prolonged ‘all-out’ maximal intensities, intensities that cause a voluntary cessation, and intensities that elicit excessive training stimulus beyond that originally intended (for more details refer to a previously published review [8]; ‘Submaximal Fitness Tests Definition’)
	*Duration*	The duration was ≤ 15 min of *exercise* for all SMFT, while e*xercise* refers to the time when HRex was monitored. Therefore, the duration of rest intervals was excluded (for example, recovery between sets)
8	Outcome Measure	HRex (expressed as %HR_max_)

*HRex* exercise heart rate, *%HR*_*max*_ percentage of HR maximum, *SMFT* submaximal fitness tests

### Screening and Study Selection

**Fig. 1:**
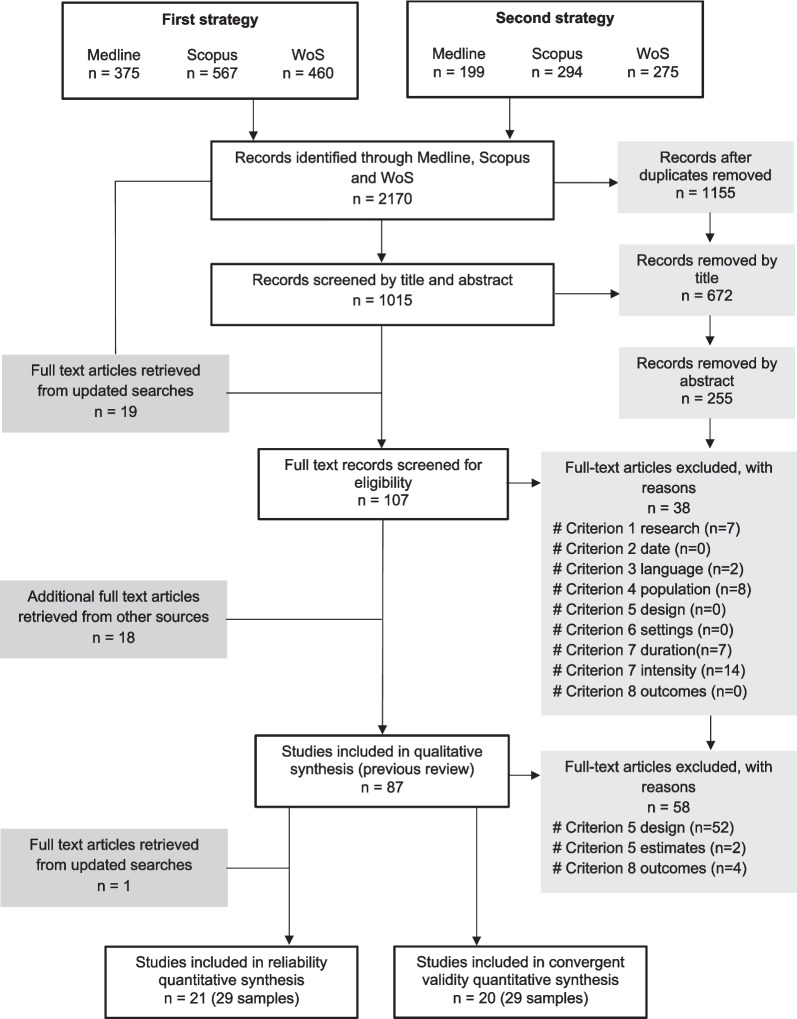
PRISMA (Preferred Reporting Items for Systematic reviews and Meta-Analyses) flow chart. The total number of studies included in the systematic review and meta-analysis was 30. 10 and 9 studies reported reliability and convergent validity effect estimates, respectively, with an additional 11 studies reporting effect estimates of both measurement properties. *n* number of studies, *WoS* Web of Science

The updated aspects in the inclusion–exclusion criteria (Table 1) for this review were related to study design and SMFT outcome measure, and the searching and screening processes are presented in Fig. 1. We sought to include studies examining SMFT HRex reliability using test–retest(s) designs and reporting absolute (group mean difference [MD], typical error of measurement [TE]) and/or relative (intraclass correlation coefficient [ICC]) effect estimates. Convergent validity studies included correlational study designs examining the relationship (correlation coefficient [*r*]) between SMFT HRex and an established team sport endurance performance test administered in a cross-sectional manner. In team sports, assessing endurance performance has been undertaken using via a variety of maximal effort and exhaustive tests administered in laboratory (typically, maximal oxygen uptake) or field-based (e.g., intermittent endurance capacity) conditions. While laboratory tests measuring maximal oxygen uptake are considered as a criterion measure of aerobic capacity, field-based tests are more pronounced in team sports (perhaps due to their practicality) and have been shown to deliver a satisfactory degree of reliability and validity [2, 21, 22]. These tests generally involve external intensity measures (either total distance covered or final running speed achieved) that are used as a proxy indicator of aerobic capacity, albeit we acknowledge other, non-aerobic qualities (anaerobic, neuromuscular) may contribute towards performance result [2, 21].

### Data Extraction and Coding

**Fig. 2:**
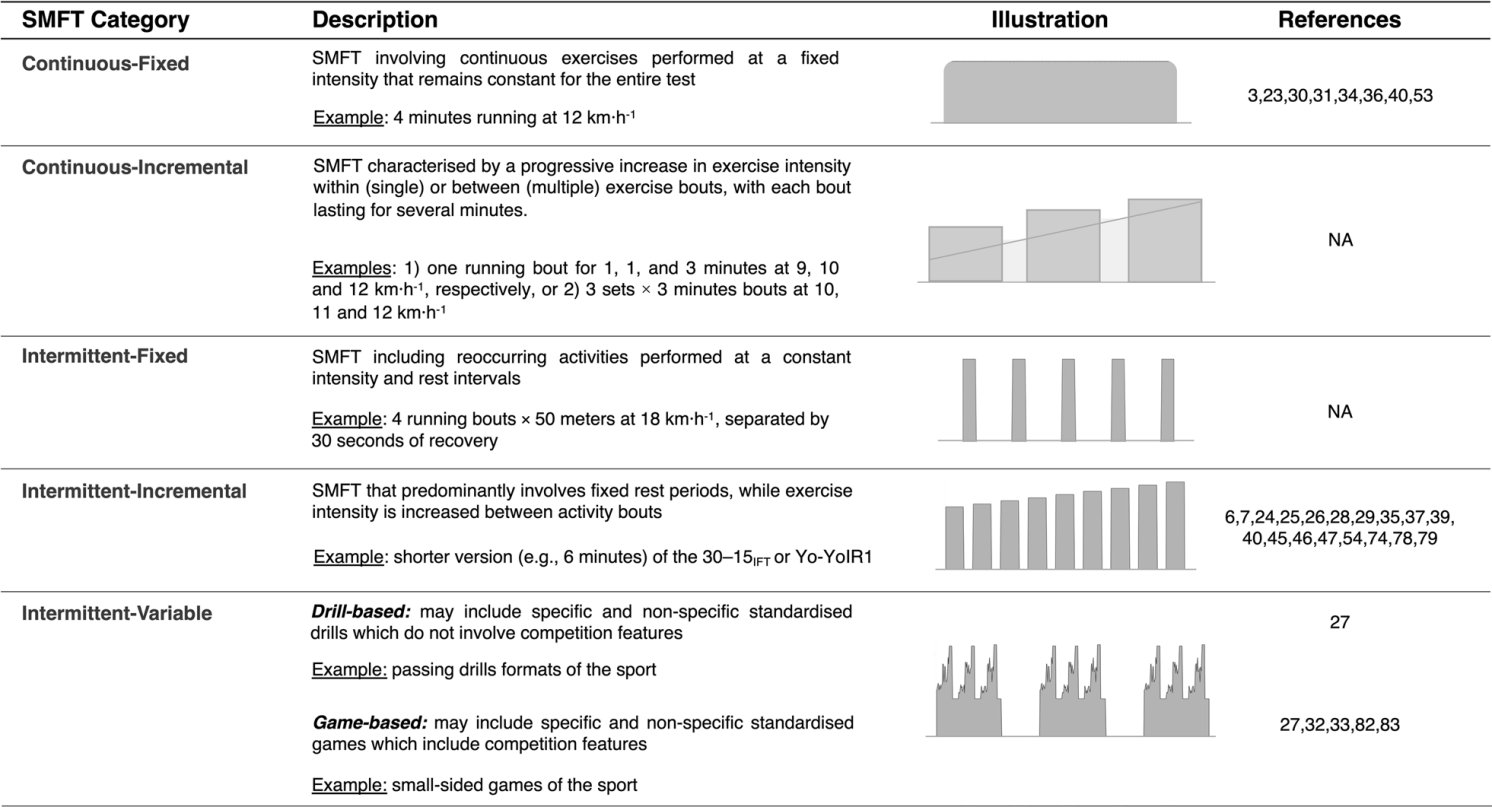
SMFT protocol categories and their utilisation in the included studies. *NA* not available

We extracted and coded the following study characteristics (Tables 2 and 3):

1. Author and year of publication.
2. Cohort mean age (years) and age category (youth [< 18 years] or senior [≥ 18 years]).
3. Sex.
4. Sport.
5. Competition level (elite, sub-elite and mixed). Based on the information provided by the authors, elite players compete in the highest level of their age group in the country. Sub-elite players do not compete in the highest level of their age group, whereas mixed represents a group of players from both levels.
6. SMFT protocol category. We earlier proposed an operational taxonomy for unique SMFT protocols based on two levels of classification, including the exercise regimen (continuous or intermittent) and rate of changes in exercise intensity (fixed, incremental or variable). Figure 2 presents the characteristics of protocols and their utilisation in the included studies.
7. SMFT duration (minutes). Based upon the details provided in the text, the total duration was rounded to the nearest 30 s.
8. SMFT internal exercise intensity, expressed as mean HRex. In our analyses, we considered HRex values analysed as percentage points of heart rate maximum (%HR_max_). Accordingly, when a study reported statistical results for both beats·min^−1^ and %HR_max_, we selected the results presented in %HRmax only. In addition, if a study reported findings solely in beats·min^−1^, we used additional information to convert the results into %HR_max_ (see “Handling Missing Data” section for details).
9. SMFT HRex collection methodology (fixed time point, mean range, or mean overall). HRex was recorded at a specific fixed time point(s) throughout the test, calculated as the mean HRex during the last during 10–60 s of the test, or calculated as the mean HRex during the overall test.
10. Reliability statistics: methodology used to calculate TE and ICC type.
11. Convergent validity (*r*): endurance performance indicator and season phase (pre-season or in-season period).

**Table 2:** Sample and SMFT characteristics of the studies included in the reliability meta-analysis

Study	Year	Sample Characteristics					SMFT						Multiple Effects	RoB (L/U/H)	Reliability Properties		
		N	Mean Age	Sex	Sport	Level	Category	Protocol	Environment	Exe Dur. (min)	Exe Int. (HRex)	Analysis			MD	TE	ICC
Veugelers et al. [46]	2016	25	23.0	Male	ARF	Elite	II	*Mod* Yo-YoIR2	Indoor AM	2–4	74.4–82.4	30 s	Vol./Int	5/1/0	✓	✓_(A)_	✓ _(3,1)_
													Test–retest				
Scott et al. [3]	2018	25	23.2	Male	Rugby	Elite	CF	Ind. Shuttle	Outdoor AM	4	81.1	30 s		6/0/0	✓	✓_(A)_	✓ _(3,1)_
									20–24 °C								
Buchheit et al. [34]	2010	18	13.9	Male	Soccer	Elite	CF	Track	Outdoor	5	81.8	30 s	Sub-groups	3/1/2	✓		
		15	15.7						21.6–23 °C		76.0						
Bradley et al. [35]	2011	32		Male	Soccer	Elite	II	Yo-YoIR2	Indoor	6	NS	Fixed		1/2/3			✓ _(NS)_
Buchheit et al. [37]	2011	12	17.0	Male	Ice Hockey	Elite	II	30-15_IFT_	Indoor	~ 3 & 6	74.6 & 88.6	20 s	Vol./Int	4/1/1	✓	✓_(A)_	✓ _(3,1)_
Fanchini et al. [7]	2014	24	17.0	Male	Soccer	Sub-elite	II	Yo-YoIR1	NS	~ 6	91.3	Fixed		3/1/2	✓	✓_(A)_	✓ _(2,1)_
Hulin et al. [6]^a^	2019	17	24.1	Male	Rugby	Elite	II	Yo-YoIR1	Outdoor	~ 4	89.0 & 91.5	10 s	Test–retest	3/2/1	✓	✓_(A)_	✓ _(3,1)_
									19.0 ± 2.6 °C								
									55 ± 13% RH								
Hulka et al. [83]	2015	25	17.7	Male	Soccer	NS	IV	4v4 SSG	Indoor	12	79.7–81.8	All		3/3/0	✓	✓_(A)_	✓ _(NS)_
Hulse et al. [26]	2013	13	U9-11	Male	Soccer	NS	II	MSFT	Indoor PM	~ 5.5	95.7	~ 60 s	Sub-groups	4/1/1	✓	✓_(B)_	✓ _(2,1)_
		15	U12-14						10.5–12.4 °C		88.7						
		18	U15-18								84.3						
Ingebrigtsen et al. [28]	2014	10–37	22.0	Male	Soccer	Mixed	II	Yo-YoIR 1/2	Indoor	2, 4 & 6	NS	Fixed	Vol./Int	1/4/1			✓ _(3,1)_
Rabbani et al. [53]	2018	12	25.1	Male	Soccer	Elite	CF	100 m shuttle	AM	4	76.7	30 s	Test–retest	5/1/0	✓	✓_(A)_	✓ _(3,1)_
									24–26 °C								
Owen et al. [29]	2017	10	17.8	Male	Soccer	Elite	II	Yo-YoIR1	Same time during the day	6	82.1	Fixed		2/4/0	✓	✓_(B)_	✓ _(2,1)_
Buchheit et al. [23]	2008	15	15.6	Male	Handball	Sub-elite	CF	Ind. Track	Indoor	6	80.5	All		3/2/1		✓_(B)_	✓ _(NS)_
									18–22 °C								
Deprez et al. [25]	2014	9–27	12.5	Male	Soccer	Mixed	II	Yo-YoIR1	Indoor	~ 2.5, 3.5 & 6.5	91.8–97.0	Fixed	Vol./Int	4/2/0	✓	✓_(B)_	✓ _(3,1)_
		8–26	14.0						Similar °C		91.5–96.7		Sub-groups				
		4–19	16.2								91.0–94.6						
Owen et al. [82]	2020	23	25.3	Male	Soccer	Elite	IV	5V5 SSG	Outdoor AM	9	87.0	All		2/2/2		✓_(A)_	✓ _(3,1)_
Doncaster et al. [54]	2018	8	12.9	Male	Soccer	Elite	II	Yo-YoIR1	Outdoor	3 & 6	88.3–93.8	15 s	Vol./Int	5/1/0	✓	✓_(A)_	✓ _(3,1)_
									11–13.2 °C								
									63–72%RH								
Deprez et al. [24]	2015	22	13.9	Male	Soccer	Elite	II	Yo-YoIR1	Outdoor PM	~ 6.5	95.1–95.4	Fixed	Sub-groups	5/1/0	✓	✓_(A)_	✓ _(NS)_
		10	16.2						~ 10 °C		91.8–92.8		Test–retest				
		4	18.1								88.1–90.0						
Dell Iacono et al. [27]^b^	2019	10	18.5	Male	Soccer	Elite	IV	Pass. Drill	Outdoor PM	12	85.1–87.2	All	Sub-groups	5/1/0	✓	✓_(A)_	✓ _(3,1)_
		10	18.7	Male	Soccer	Elite	IV	5V5 SSG	22 ± 2.5 °C	12	86.2–87.1						
									65 ± 3.8%RH								
Younesi et al. [33]	2021a	16	27.2	Male	Soccer	Elite	IV	3V3-6V6 SSG	Outdoor PM	6	80.8–81.8	All	Protocol	5/1/0	✓	✓_(A)_	✓ _(3,1)_
									25–32 °C				Test–retest				
									54–76%8RH								
Ryan et al. [31]	2019	16	24.6	Male	ARF	Elite	CF	80 m shuttle	Outdoor	5		30 s		4/2/0	✓	✓_(A)_	✓ _(3,1)_
									20–25.5 °C								
Scott et al. [36]^c^	2022	17	22.0	Male	Rugby	Elite	CF	Ind. Shuttle	21–23 °C	4	84.9	30 s		5/1/0	✓	✓_(A)_	✓ _(3,1)_

*30-15*_IFT_ 30-15 intermittent fitness test, *AM* morning hours, *ARF* Australian football, *All* overall exercise, *Fixed* fixed time point(s), *°C* temperature in Celsius, *CF* continuous fixed, *Exe.* Exercise, *H* high, *ICC* intraclass correlation, *II* Intermittent incremental, *Ind.* Individualised, *Int* intensity, *IV* intermittent variable, *L* low, *m* metres, *MD* mean difference, *MSFT* multistage fitness test, *N* sample size, *NS* not specified, *PM* afternoon hours, *Pass* passing, *RH* relative humidity, *RoB* Risk of Bias*, SSG* small-sided games, *TE* typical error of measurement, *U* unclear, *s* second, *SMFT* submaximal fitness test, *Vol* volume, *Yo-YoIR1* Yo-Yo intermittent recovery test level 1, *Yo-YoIR2* Yo-Yo intermittent recovery test level 2, *mod. Yo-YoIR2* modified (18 m shuttle) Yo-Yo intermittent recovery test level 2. TE_(A)_: TE derived from the mean variance of individuals score change (Eq. 2), TE_(B)_: TE derived from the variance between individuals and ICC (Eq. 3). ICC _(2,1)_ and ICC _(3,1)_ refer to ICC type

^a^Test–retest reliability results were established using the raw data provided by the authors

^b^Test–retest reliability results of the first two weeks which met the inclusion criteria of total duration were established using the raw data provided by the authors

^c^Test–retest reliability results were established using the raw data provided by the authors

**Table 3:** Sample and SMFT characteristics of the studies included in the convergent validity meta-analysis

Study	Year	Sample Characteristics					SMFT						Multiple effects	RoB (L/U/H)	Performance Criterion
		N	Mean age	Sex	Sport	Level	Category	Protocol	Season phase	Exe Dur. (min)	Exe Int. (HRex)	Analysis			
Veugelers et al. [46]	2016	38	23.0	Male	ARF	Elite	II	Yo-YoIR2	Pre-season	2–8	76.4–92.8	30 s	Vol./Int	5/1/0	Yo-YoIR2 (m)
								Mod							
								Yo-YoIR2							
Mohr & Krustrup [45]	2014	172	25.8	Male	Soccer	Sub-elite	II	Yo-YoIR1	Pre & in-season	6	85.9–90.9	Fixed	Repeated Measures	2/3/1	Yo-YoIR1 & 2 (m)
Bradley et al. [35]	2011	32		Male	Soccer	Elite	II	Yo-YoIR2	NS	4 & 6	87.0 & 91.5	Fixed	Vol./Int	1/4/1	Yo-YoIR2 (m)
		14											Sub-groups		
Bradley et al. [74]	2014	28	23.0	Female	Soccer	Elite	II	Yo-YoIR2	NS	2 & 4	90.0–96.0	Fixed	Vol./Int	2/4/0	Yo-YoIR2 (m)
Fanchini et al. [7]	2014	24	17.0	Male	Soccer	Sub-elite	II	Yo-YoIR1	Pre & in-season	6	90.3–93.9	Fixed	Repeated Measures	2/3/1	Yo-YoIR1 (m)
Ingebrigtsen et al. [79]	2012	39	20.0	Male	Soccer	Sub-elite	II	Yo-YoIR1/2	In-season	2 & 4	91.1–97.8	Fixed	Vol./Int	2/2/2	Yo-YoIR1 and 2 (m)
		12	26.0			Elite					80.8		Protocol		
													Sub-groups		
Ingebrigtsen et al. [28]	2014	57	22.0	Male	Soccer	Mixed	II	Yo-YoIR1	In-season	2 & 4	NS	Fixed	Vol./Int	1/4/1	Yo-YoIR1 and 2 (m)
													Protocol		
Lignell et al. [47]	2018	18	28.0	Male	Ice Hockey	Elite	II	Yo-YoIR1	In-season	6	78.9	Fixed		3/3/0	VO_2_ max (ml·min^−1^·kg^−1^)
Rabbani et al. [53]	2018	14	26.7	Male	Soccer	Elite	CF	100 m shuttle	In-season	4	76.7	30 s		5/1/0	30-15_IFT_
															(km·h^−1^)
Vigh-Larsen et al. [39]	2019	245	21.7	Male	Ice Hockey	Mixed	II	Yo-YoIR1	In-season	6	83.3	60 s		3/3/0	Yo-YoIR1 (m)
Hulin et al. [6]	2019	32	24.1	Male	Rugby	Elite	II	Yo-YoIR1	Pre-season	~ 4 & 6	89.8 & 92.8	10 s	Vol./Int	5/1/0	Yo-YoIR1 (m)
Francini et al. [40]	2019	68	14.5	Male	Soccer	Sub-elite	CF	Mognomi’s	In-season	6	95.4	60 s	Protocol	3/3/0	Yo-YoIR1 (m)
							II	Yo-YoIR1			94.4	Fixed			
Pereira et al. [30]	2019	40	11.5	Male	Soccer	NS	CF	5′–5′ 20 m rectangle	NS	5	78.4	30 s		5/1/0	Yo-YoIR1 (m)
Buchheit et al. [34]	2010	33	14.7	Male	Soccer	Elite	CF	Track	In-season	5	78.4	30 s		3/3/0	Vam-Eval (km·h^−1^)
Owen et al. [82]	2020	23	25.3	Male	Soccer	Elite	IV	5V5 SSG	In-season	9	87.0	All		3/2/1	Yo-YoIR1 (m)
Araújo et al. [78]	2019	16	24.0	Male	Soccer	Elite	II	ISRT	In-season	2, 4, 6 and 9	74.0–91.0	Fixed	Vol./Int	4/2/0	ISRT (m)
		14	24.0	Male		Sub-elite					77.0–92.0		Sub-groups		
		16	21.0	Female		Elite					83.0–97.0				
		15	25.0	Female		Sub-elite					88.0–97.0				
Deprez et al. [25]	2014	19	12.5	Male	Soccer	Mixed	II	Yo-YoIR1	In-season	~ 6.5	97.0	Fixed	Sub-groups	2/2/2	Yo-YoIR1 (m)
		18	14.0								96.7				
		4	16.2								94.6				
Deprez et al. [24]	2015	22	13.9	Male	Soccer	Elite	II	Yo-YoIR1	NS	~ 6.5	95.3	Fixed	Sub-groups	4/1/1	Yo-YoIR1 (m)
		10	16.2	Male	Soccer	Elite					92.3				
		4	18.1	Male	Soccer	Elite					89.2				
Younesi et al. [32]^a^	2021b	16	27.2	Male	Soccer	Elite	IV	3V3 SSG	Pre-season	6	80.1–80.8	All	Repeated Measures	3/2/1	30-15_IFT_ (km·h^−1^)
Scott et al. [36]^b^	2022	12	24.0	Male	Rugby	Elite	CF	Ind. Shuttle	Pre-season	4	83.2	30 s		5/1/0	30-15_IFT_ (km·h^−1^)

*30-15*_IFT_ 30-15 intermittent fitness test, *ARF* Australian football, *All* overall exercise, *Fixed* fixed time point(s), *CF* continuous fixed,* H* high, *II* Intermittent incremental, *Int* intensity, *ISRT* interval shuttle run test, *IV* intermittent variable, *L* low, *N* sample size, *NS* not specified, *RoB* Risk of Bias, *U* unclear, *s* second, *SMFT* submaximal fitness test, *Vol* volume, *Yo-YoIR1* Yo-Yo intermittent recovery test level 1, *Yo-YoIR2* Yo-Yo intermittent recovery test level 2, SSG small-sided games

^a^Correlation coefficient of each independent session were provided by the authors

^b^Exercise intensity value was established using the raw data provided by the authors

### Handling Missing Data

We used direct contact details of the corresponding author(s), along with social networks (e.g., ResearchGate, Twitter) to attain missing information including: (1) sample characteristics (e.g., mean age, sample size); (2) SMFT characteristics (duration, exercise or drill/game configuration, etc.); (3) outcome measures, including HRex values recorded during the SMFT, or sample HR_max;_ and (4) other relevant reliability and/or convergent validity statistical calculation methods or results. Overall, we contacted 12 authors (15 studies) [3, 6, 23–35] for additional information, and 9 (12 studies) of them replied [3, 6, 23–27, 29, 31–34], with most providing explanations [23–25, 29, 31, 32, 34] or the entire raw data [3, 6, 27, 36]. The remaining information was either excluded from pooled effect estimates analyses, obtained from alternative details provided within the text, or omitted when a meta-regression analysis was performed. All data estimations employed were considered in the qualitative assessment of individual studies (see “Study Qualitative Assessment” section.

In some cases, the original sample size reported in the study was modified when reliability or convergent validity experiments were administered. When the new characteristics (as an example, mean age) relating to the modified sample were not reported, the original information provided was used. With regards to the reliability dataset, two studies [7, 37] that collected SMFT HRex in beats·min^−1^ also reported HR maximum derived from the maximal version of the same test, meaning the conversion into relative values was straight-forward. One study [26] analysed SMFT HRex similarly (beats·min^−1^) and did not report HR_max._ In addition, ages were reported as U9–U11, U12–U14 and U15–U18. In this case, HR_max_ was calculated using an estimated equation (HR_max_ = 208 – 0.7 × mean age [38]), and mean ages were assumed as 9, 12 and 16 years, respectively. Similar HR_max_ estimation was employed in two previous studies [29, 33]. Graph digitizer software (WebPlotDigitizer, https://apps.automeris.io/wpd/) was used to extract the first two SMFT HRex group means and standard deviations of a test–retest study that administered repeated measures [34].

With respect to the convergent validity dataset, in two studies [7, 30] using SMFT HRex as beats·min^−1^, one [7] reported HR_max_, and for the other [30] we estimated HR_max_ as described above. One study [35] reported age as youth U19, the estimated mean age was 17, and the same graph digitizer software was used to extract the mean SMFT exercise intensity (HRex). Further, three studies [34, 39, 40] combined sub-groups when convergent validity was analysed. In that case, the weighted mean SMFT HRex and age of the sub-groups were calculated for exercise intensity and mean age, respectively.

### Effect Estimates

#### Mean Difference

The Mean Difference (MD) is an absolute reliability estimate which indicates the degree of variability relating to systematic, group-level bias (i.e., total sample) [15]. As discussed in previous sections, we considered MD effect estimates presented raw units (%HR_max_), which were calculated as the change in group means across test–retest [41].

#### Typical Error of Measurement

Another absolute reliability estimate is Typical Error of Measurement (TE) [15, 16]. The TE is an indicator of random, within-subject variability and is sometimes presented in the form of coefficient of variation (CV%) [15]. This approach is appealing for normalization and interpretation of variability, especially across different outcome measures. However, since the primary outcome measure in our meta-analysis is already analysed in relative values (%HR_max_), it is not appropriate (or interpretable) to present TE as a CV (i.e., percentage of percentage). Therefore, when a study presented the TE as a CV value, we estimated the former using the following equation:

1 $$\left( {\frac{{{\text{CV}}}}{100}} \right) \times {\text{grand}}\;{\text{mean}}\;{\text{HRex}}$$

where grand mean is the mean HRex across test 1 and test 2.

Two common methods for calculating TE are cited in the sport science literature [15, 16], and were identified in our review. The first approach proposed by Atkinson and Nevill [16] uses the ICC in combination with the pooled between-athlete variance (Eq. 2). The second method proposed by Hopkins [42] uses the mean variance of within-athlete change scores (Eq. 3). Since the former approach requires the sample standard deviation and ICC, it is usually easier to extract from an article. However, after performing different simulations with our previous (unpublished) work and the available data in our included studies, and generating fictitious data with similar values, we found that the difference in TE calculated using both methods negligible to almost non-existent (available in the Additional file 3), suggesting that it is appropriate to meta-analyse estimates calculated from either method without any transformation. If the exact method was not specified nor provided after personal communication to the author, or TE was not disclosed in the analysis, we used the Atkinson and Nevill [16] method (i.e., Eq. 2), where possible [35].

2 $${\text{TE}} = {\text{SD}} \times \sqrt {1 {-} {\text{ICC}}}$$

3 $${\text{TE}} = \frac{{{\text{SD}}_{\Delta } }}{{\sqrt {2} }}$$

where SD_Δ_ is the standard deviation of individual change scores (test 2–test 1% point) and SD is the pooled between-athlete standard deviation observed in test 1 and test 2.

#### Intraclass Correlation Coefficient

The ICC represents reproducibility in the rank order of athletes over repeated measures (i.e., relative reliability) [43]. In most of our eligible studies, two types of ICC were identified: those describing absolute agreement in a single measure from a two-way random effects model (ICC_2,1_), and those describing consistency in a single measure from a two-way mixed-effects model (ICC_3,1_) [43], whereas in other studies ICC type was not clearly specified or attainable (Table 2). Based on the comparisons used in the TE data synthesis, the differences between ICC types were negligible (Additional file 3), and we thereby treated all data the same. We acknowledge that there are conceptual differences between ICC types [43]. However, it was not possible to examine this effect due to the low number of estimates in some levels (e.g., ICC_2,1_, *n* = 3 studies).

#### Pearson’s Correlation Coefficient (*r*)

We elected to extract Pearson’s product-moment correlation coefficient (*r*) as the principal statistic estimate to describe the magnitude of association between SMFT HRex and maximal endurance performance [44]. As demonstrated in Table 3, most reference tests included field-based, intermittent incremental protocols with change of direction (COD) such as the Yo-Yo Intermittent Recovery Test Level 1 (Yo-YoIR1) [45] or 2 (Yo-YoIR2) [46], while other individual studies involved continuous incremental protocols, including the Vam-Eval test [34] and maximal oxygen uptake in laboratory conditions [47].

### Statistical Methods and Data Analysis

#### Synthesis of Effect Estimates Prior and Post Analysis

An overview of calculations for effect estimates and their sampling variance is presented in Table 4. The MD estimate and its sampling variance were calculated from the test–retest statistics of sample size (n), group means and standard deviations while assuming equal sampling variances within groups [41]. The ICC and *r* estimates and their sampling variances were converted to Fisher’s *z* values to approximate normally distributed data [41, 48], and subsequently back-transformed for analyses interpretations of the pooled estimates (intercept-only model). To our knowledge, there is no documented approach for a meta-analysis of TE. Therefore, we used the method proposed by Nakagawa et al. [49] for meta-analysis of variation. This method uses a log-transformed standard deviation (adjusted for sample size), along with its sampling variance for each individual group [49]. For obtaining the pooled estimate, the results were back-converted to their original values, including the bias correction for sample size. It is important to note that considering that the transformation of meta-regression coefficients (full-model) back into their original values is not straight-forward and may result in erroneous interpretations of the modifying effects, coefficients are presented in Fisher’s *z*-scale (ICC and *r*) and log-transformed (TE) values. To facilitate models' interpretations, we constructed meta-regression bubble plots, presenting the predicted values and their corresponding confidence and prediction interval bounds.

**Table 4:** Effect estimates and their sampling variances

Primary outcome	Effect estimate		Sampling variance
MD	Raw values	${\text{MD}} = M_{1} - M_{2}$	$V_{{{\text{MD}}}} = \frac{{n_{1} + n_{2} }}{{n_{1} n_{2} }}S_{{{\text{pooled}}}}^{2}$ where $S_{{{\text{pooled}}}} = \sqrt {\frac{{\left( {n_{1} - 1} \right)S_{1}^{2} + \left( {n_{1} - 1} \right)S_{2}^{2} }}{{n_{1} + n_{2} - 2}}}$
TE	Log-transformed	${\text{TE}} = \ln s + \frac{1}{{2\left( {n - 1} \right)}}$	$V_{TE} = 1/2\left( {n - 1} \right)$
ICC	Fisher’s *z*	$z = 0.5{ } \times {\text{ln}}\left( {\frac{1 + r}{{1 - r}}} \right)$	$V_{icc} = 1/\left( {n - 3/2} \right)$
*r*	Fisher’s *z*	$z = 0.5{ } \times {\text{ln}}\left( {\frac{1 + r}{{1 - r}}} \right)$	$V_{r} = 1/\left( {n - 3} \right)$

*ICC* intraclass correlation coefficient*, ln* natural logarithm (log-transformed), *M*_*1*_ group mean test 1, *M*_*2*_ group mean test 2, *MD* mean difference, *n*_*1*_ and *n*_*2*_ sample size, *r* correlation, *TE* typical error of measurement, *s TE*, *S*_*1*_ group standard deviation test 1, *S*_*2*_ group standard deviation test 2, *V*_TE_ typical error of measurement sampling variance, *V*_*icc*_ intraclass correlation sampling variance; *V*_*r*_ correlation coefficient sampling variance; *z* Fisher’s *z*. ICC sampling variance was adjusted following previous recommendations (48)

#### Overall Meta-Analysis

All data analyses were conducted using the *metafor* [50] and *clubSandwich* [51] packages in the R studio environment (version 1.4.1106) [52]. The datasets and analysis codes are openly available in the Additional files (https://osf.io/mqnt9/). In most of the included studies, we were able to extract more than a single effect size. Multiple effect sizes were derived from different sub-groups within studies, but more frequently from a variety of SMFT characteristics (e.g., SMFT protocols, duration or intensities) within sub-groups. In addition, some of the reliability studies [6, 24, 46, 53, 54] included more than two similar consecutive SMFT and provided the results from each matched test–retest. Two studies examining convergent validity [7, 45] implemented within-group repeated measures of the same tests at different time-points across the season (Table 2 and 3 provide the sources of multiple effect estimates within studies).

Given the hierarchical structure in our datasets (multiple effect estimates are nested within groups), as well as the likelihood of statistical dependency, we employed a more recently developed approach using multilevel mixed-effects meta-analysis and robust variance estimation with adjustments for small-samples [55]. This approach allows exploration of the variance present across multiple levels and, hence, within- and between-group variance [56], and provides a robust method for meta-analysis while accounting for the dependency among effect estimates derived from common samples [57]. In such cases, replacing sampling variance with the entire ‘V matrix’, indicating the variance–covariance matrix of the estimates, can further account for the correlation between effect estimates [55, 58]. As it was not possible to attain the correlation between effect estimates drawn from the same participants in most of the included studies, our analyses were conducted using an assumed constant correlation of *ρ* = 0.6 [55]. In Additional file 4: Table S3 we report sensitivity analyses, whereby a range of correlation values were used to evaluate the influence of the changes in the within-group covariance on the pooled estimates and its variance components. Collectively, these analyses showed identical pooled estimates and nearly similar variance components.

#### Heterogeneity and Modifying Effects

To describe the extent of heterogeneity, we calculated restricted maximum likelihood estimates of the within- and between-group variances (SD; tau [*τ*]) [59], as well as the *I*^2^ statistic in each level [60] which implies the percentages of variance which are due to study heterogeneity rather than sampling error [60]. Log-likelihood-ratio tests were also computed to determine whether the within- and between-group variances were significant [61]. We then conducted regression analyses to examine possible source of heterogeneity, including parameters related to SMFT (e.g., exercise intensity) or athlete’s (e.g., mean age) characteristics. Modifying effects with at least 6 independent samples and overall 10 effect estimates in each level [62] were added separately to the models. These were analysed independently, rather than in combination, due to a wide range of sample sizes for different modifying effects that resulted in a relatively low number of estimates for the requirement of meta-regression [62].

Categorical effects were evaluated as the differences between levels and consisted of athlete’s age category (youth, senior), HRex collection method (reliability: fixed, mean range, mean overall; convergent validity: fixed, mean range) and competition level (elite, sub-elite; for convergent validity only). Continuous effects included athlete’s mean age (years), SMFT exercise intensity (expressed as HRex) and SMFT duration (minutes), and were explored as the changes associated with pre-defined clinically relevant values: (1) for mean age, we explored the effect of 3-year change in age; (2) SMFT exercise intensity effect was evaluated as the changes associated with 5-point % change in HRex; and (3) the effect of SMFT duration was investigated in reference to 2-min change in duration. Finally, model strength was assessed as the percentage of variance explained by the modifying effect with pseudo-*R*^2^ statistic (i.e., intercept-only versus full-model) [41]. When variances in both levels were identified, we scrutinised *R*^2^ in the within (*R*^2^_2_) and between (*R*^2^_3_) group variance separately [56].

#### Inferences

Uncertainty in meta-analysis and regression estimates was expressed using 95% compatibility (confidence) intervals (CI), representing ranges of values compatible with our models and assumptions [63]. We also derived 95% prediction intervals (PI), which convey the likely range of the true measurement properties in similar future studies. Similar to others [64], we sought to avoid dichotomizing the presence or absence of an effect using traditional null hypothesis testing. Instead, we considered the entire range of 95% CI, relating mostly to the point estimate and uncertainty of predicted values. Effects were then discussed in terms of their compatibility (or coverage) with practically significant or practically equivalent values. Qualitative inferences were made for standardised effects only (i.e., ICC and *r)* using thresholds of > 0.99, extremely high; 0.90–0.99, very high; 0.75–0.90, high; 0.50–0.75, moderate; 0.20–0.50, low; < 0.20, very low for ICC [65] and 0.10 small; 0.30 moderate; 0.50 large; 0.70 very large; and 0.90 extremely large for *r* [66]. Thresholds for the smallest meaningful correlation (i.e., 0.1 and 0.2 for *r* and ICC, respectively) were used to declare modifying effects as substantial [44]. We elected to not make formal decisive interpretation on the substantiality of MD and TE because these are presented in units of the outcome measure (i.e., HRex), and we are as yet unaware of the true, minimum practically important difference for team sport athletes during SMFT.

### Study Qualitative Assessment

Qualitative assessment of individual studies was conducted using the Risk of Bias Assessment Tool for Non-randomised Studies (RoBANS) [67, 68]. The RoBANS comprises six-dimension criteria which were adapted to answer questions that may influence the reliability or convergent validity results reported in the included studies. Answer categories of ‘low’, ‘unclear’ and ‘high’ risk of bias were assigned in each domain. To promote consistency in how domains were evaluated, three reviewers (TS, RL, SJM) created a decision rule for each criterion. Assessments were then performed by one reviewer (TS), with two other authors (RL, SJM) checking for accuracy. A summary detailing the quality assessment criteria in each domain is provided as Additional file 5: Table S1.

### Small-Study Effects and Influence Analysis

Small-study effects and asymmetry were visually inspected using funnel plots [69]. To confirm our visual impression, the Begg’s rank correlation [70] and Egger’s regression (by fitting the square root of the sampling variance as a moderator) [69] tests were employed. Further, to assess the influence of each effect size on the summary effect and heterogeneity, Cook’s distance analysis [71] and Baujat plots [72] were obtained. A standard rule of thumb is that Cook distance values greater than three times the mean were used [73], and potential outliers were excluded from the dataset to examine if the change in the summary estimates turned substantial.

## Results

### Selected Reliability Studies and Characteristics

Table 2 summarises the characteristics of included reliability studies. The overall dataset included 29 samples derived from 21 studies. The MD dataset consisted of 69 effect sizes nested within 25 samples (mean = 2.8 [effect sizes per sample], median = 2, range of 1–24), derived from 18 studies. The TE dataset included 52 effect sizes nested within 25 samples (mean = 2.1, median = 1, range of 1–12), from 18 studies. The ICC dataset included 54 effect sizes nested within 27 samples (mean = 2.0, median = 1, range of 1–12), from 20 studies. The total number of team sport athlete inclusions was 406 in MD, 411 in TE and 480 in ICC datasets. The studies involved male players only, and the majority originated from soccer (76%), followed by rugby codes (10%), Australian football (7%), ice hockey and handball (one study). Elite players were included in 76% of the studies, with a further 16% being mixed and 8% being sub-elite. The proportion of youth and senior athletes was 55% and 45%, respectively. SMFT categories were distributed as follows: intermittent incremental (59%), continuous fixed (24%) and intermittent variable (17%).

### Selected Convergent Validity Studies and Characteristics

Table 3 summarises the characteristics of our included convergent validity studies. The overall dataset included 73 effect sizes nested within 29 samples (mean = 2.5, median = 2, range of 1–10), derived from 20 studies, with a total number of 1055 team sport athlete inclusions. Most studies (90%) involved male players, and the majority originated from soccer (83%), ice hockey and rugby codes (6.5% each) and Australian football (one study). Elite players were included in 61% of the studies, with a further 21% being sub-elite and 18% being mixed. Unlike reliability, 62% and 38% of studies were conducted on senior and youth athletes, respectively. Most (73%) studies were conducted during the in-season phase, and the distribution of SMFT categories was similar: intermittent incremental modality (76%), continuous fixed (17%) and intermittent variable (7%).

### Overall Meta-Analyses

Table 5 provides a summary of the reliability and convergent validity meta-analyses, with forest plots of the weighted points estimates, 95% CI and PI presented in Additional file 6: Fig. S1–4. The pooled estimates derived from the reliability analyses indicated overall good absolute and high relative reliability. The pooled estimate derived from *r* dataset indicated an inverse, large relationship between SMFT HRex and endurance performance measures.

**Table 5:** Meta-analysis of measurement properties

Measurement property	Statistic	Number of		Pooled Effect		
		Studies	Estimates	Estimate	95%CI (lower to upper)	95%PI (lower to upper)
Reliability	MD	25	69	0.5	0.1 to 0.9	0.1 to 0.9
	TE	25	52	1.6	1.4 to 1.9	0.9 to 3.0
	ICC	27	54	0.88	0.84 to 0.91	0.59 to 0.97
Convergent validity	*r*	29	73	–0.58	– 0.62 to − 0.54	− 0.77 to − 0.31

*CI* confidence intervals, *ICC* intraclass correlation coefficient, *MD* mean difference, *PI* prediction intervals, *TE* typical error of measurement, *r* correlation coefficient

### Heterogeneity

We found no evidence for heterogeneity in the MD meta-analysis (*τ*_2_ = 0.09 [95% CI 0.00 to 0.49], *τ*_3_ = 0.00 [95% CI 0.00 to 0.72], *I*^*2*^_2_ = 0.5% [95% CI 0.0% to 14.2%] and *I*^*2*^_3_ = 0% [95% CI 0.0% to 26.4%]) and therefore did not employ meta-regression analysis. In contrast, notable heterogeneity was observed in all other reliability estimates: TE (*τ*_2_ = 0.23 [95% CI 0.15 to 0.32], *τ*_3_ = 0.18 [95% CI 0.00 to 0.34], *I*^*2*^_2_ = 43.4% [95% CI 42.8% to 76.8%], *I*^*2*^_3_ = 28.4% [95% CI 0.0% to 78.3%]), and ICC (*τ*_2_ = 0.26 [95% CI 0.18 to 0.36], *τ*_3_ = 0.20 [95% CI 0.00 to 0.40], *I*^*2*^_2_ = 39.4% [95% CI 35.0% to 68.3%], *I*^*2*^_3_ = 24.4% [95% CI 0.0% to 72.5%]). A greater source of within- versus between-group variance was evident in both TE and ICC. Heterogeneity in *r* was present in the within-group level only (*τ*_2_ = 0.16 [95% CI 0.11 to 0.21], *τ*_3_ = 0.00 [95% CI 0.00 to 0.14], *I*^*2*^_2_ = 48.3% [95% CI 33.0% to 62.9%], *I*^*2*^_3_ = 0% [95% CI 0.0% to 42.6%]).

### Meta-Regression Analysis

**Fig. 3:**
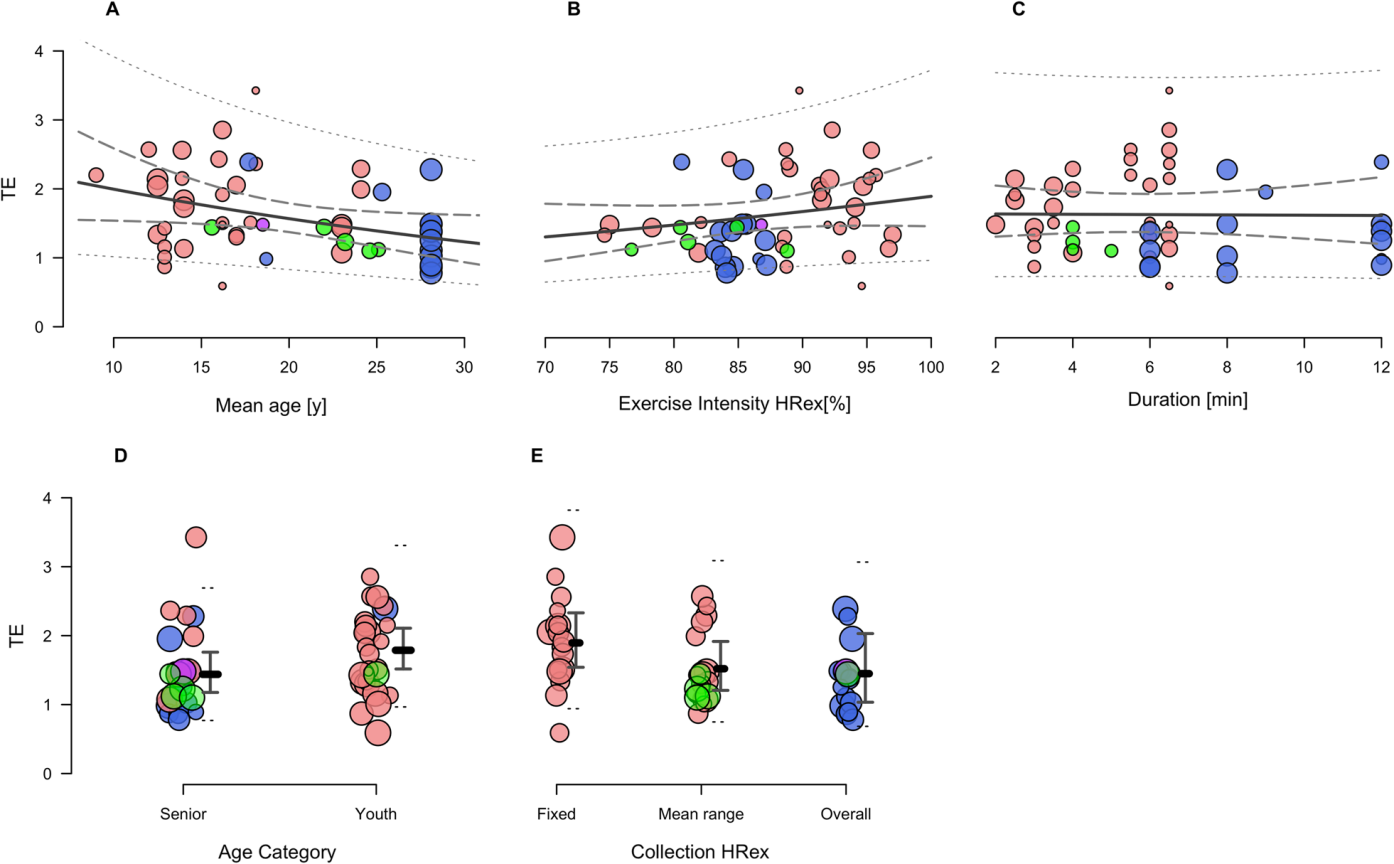
Mixed-effects meta-regression of the changes in Intraclass Typical Error of measurement (TE) while controlling for the effects of mean age (**A**); SMFT exercise intensity (HRex) (**B**); SMFT duration (**C**); age category (senior vs. youth) (**D**); and HRex collection method (fixed vs. mean range vs. mean overall) (**E**). Data points represent individual effect estimates included in our meta-analysis, and the size of the data point is proportional to their weighting. Green, red, blue and purple points represent studies using continuous fixed, intermittent incremental, intermittent variable small-sided games, and intermittent variable passing drills category, respectively. Solid lines represent the estimate of the modifying effect. Dashed and dotted lines represent 95% confidence and prediction limits, respectively. *HRex* exercise heart rate, *y* years, *min* minutes

**Fig. 4:**
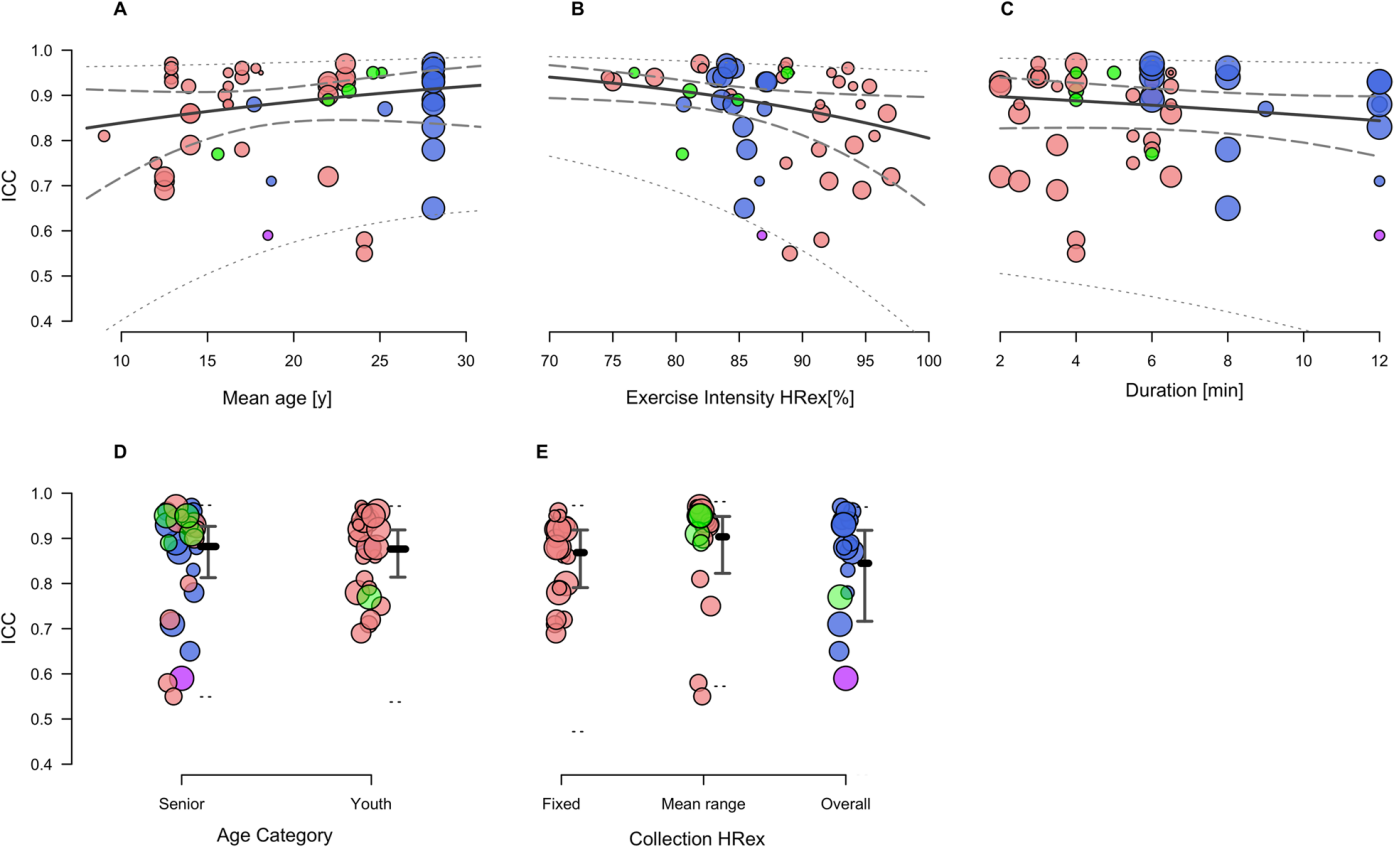
Mixed-effects meta-regression of the changes in Intraclass Correlation Coefficient (ICC) while controlling for the effects of mean age (**A**); SMFT exercise intensity (HRex) (**B**); SMFT duration (**C**); age category (senior vs. youth) (**D**); and HRex collection method (fixed vs. mean range vs. mean overall) (**E**). Data points represent individual effect estimates included in our meta-analysis, and the size of the data point is proportional to their weighting. Green, red, blue and purple points represent studies using continuous fixed, intermittent incremental, intermittent variable small-sided games, and intermittent variable passing drills category, respectively. Solid lines represent the estimate of the modifying effect. Dashed and dotted lines represent 95% confidence and prediction limits, respectively. *HRex* exercise heart rate, *y* years, *min* minutes

**Fig. 5:**
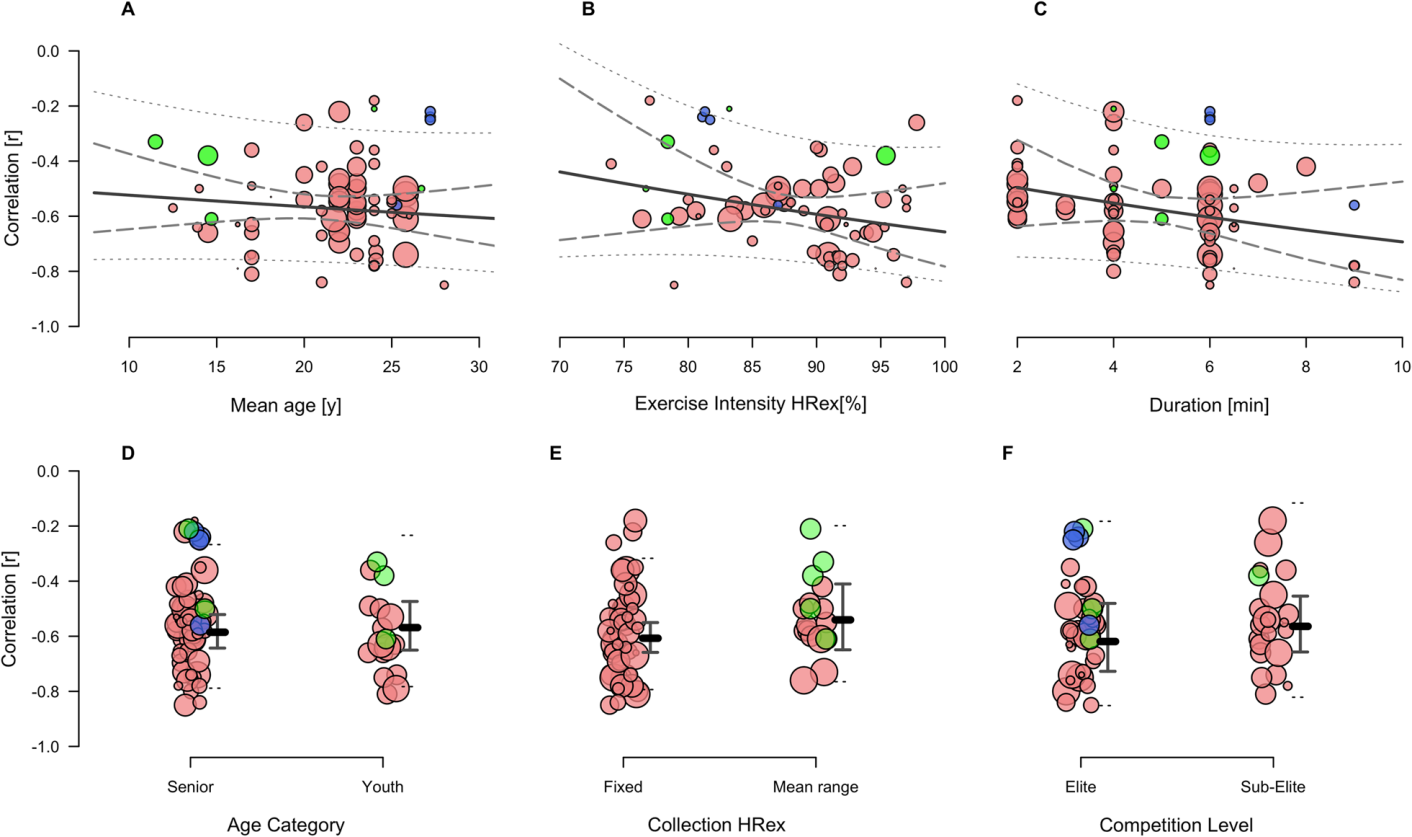
Mixed-effects meta-regression of the changes in Correlation Coefficient (*r)* while controlling for the effects of mean age (**A**); SMFT exercise intensity (HRex) (**B**); SMFT duration (**C**); age category (senior vs. youth) (**D**); HRex collection method (fixed vs. mean range) (**E**); and level (elite vs. sub-elite) (**F**). Data points represent individual effect estimates included in our meta-analysis, and the size of the data point is proportional to their weighting. Green, red and blue points represent studies using continuous fixed, intermittent incremental and intermittent variable modality, respectively. Solid lines represent the estimate of the modifying effect. Dashed and dotted lines represent 95% confidence and prediction limits, respectively. *HRex* exercise heart rate, *y* years, *min* minutes

Figures 3, 4 and 5 illustrate meta-regression bubble plots of the modifying effects, with different colours indicative of SMFT category. The full meta-regression results are presented in Table S2 in Additional file 4. The TE regression coefficients ranged between ln_diff_ = − 0.27 to 0.06, which were approximately equal to differences of 0.01 to 0.45 when comparing between TE predicted values (Fig. 3, panel A–E). The ICC regression coefficients ranged between *z*_diff_ = − 0.10 to 0.16 and resulted in approximate differences of 0.00 to 0.03 between ICC predicted values (Fig. 4, panel A–E). The *r* regression coefficients ranged between *z*_diff_ = − 0.08 to 0.10, indicating approximate correlation differences of − 0.07 to − 0.02 between *r* predicted values (Fig. 5, panel A–F). Collectively, none of these modifying effects appeared to have a meaningful influence on the reliability or convergent validity estimates. There was, however, a considerable reduction in the overall heterogeneity owing to the inclusion of some of the variables.

### Study Qualitative Assessment

**Fig. 6:**
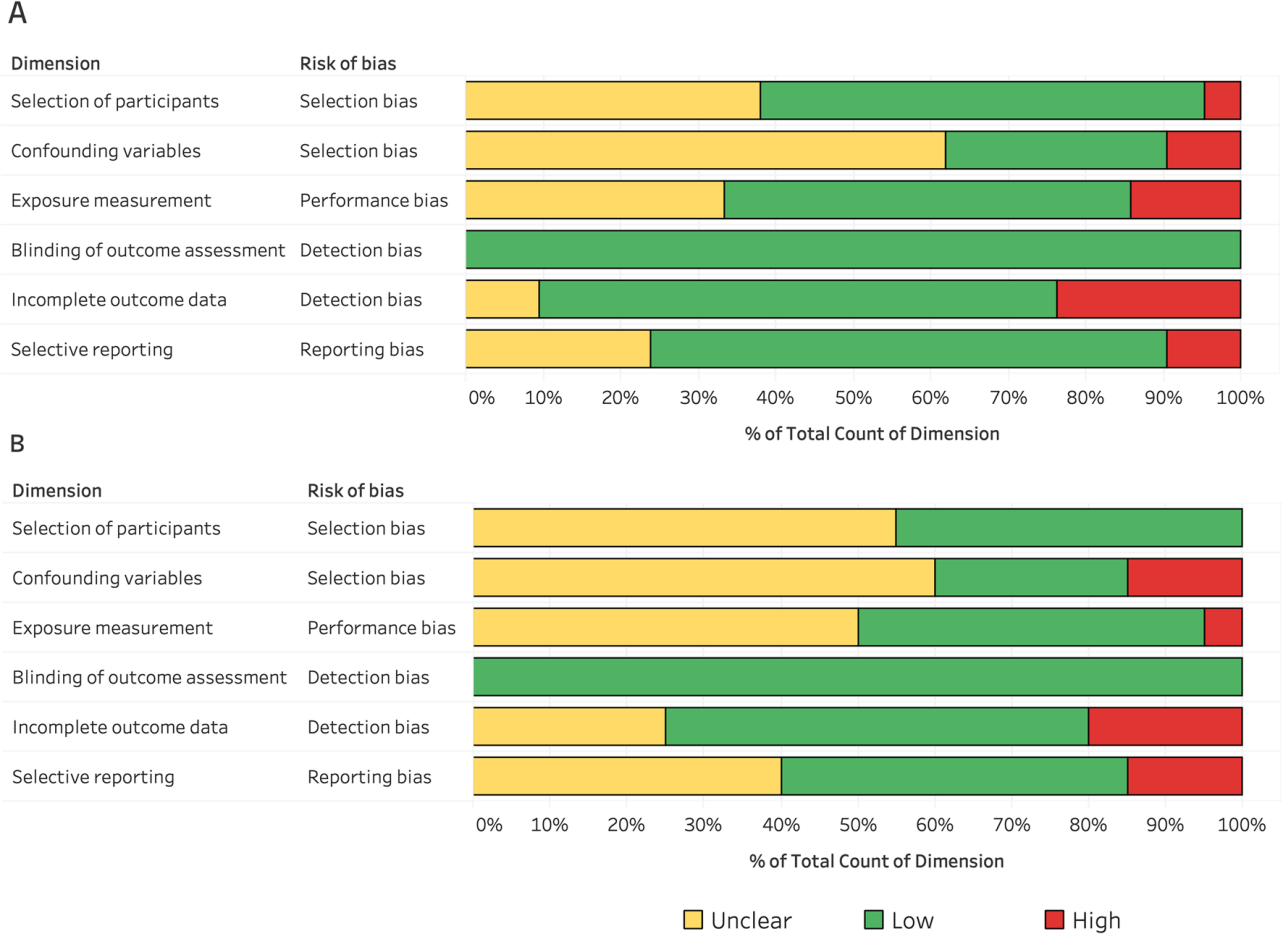
Distribution of Risk of Bias for reliability (**A**) and convergent validity (**B**) included studies

Risk of bias assessment is demonstrated in Tables 2 and 3, including the sum of each risk category (low, unclear or high) in individual articles. A graphical overview of the overall risk in each domain from all studies is illustrated in Fig. 6.

### Small-Study Effects

**Fig. 7:**
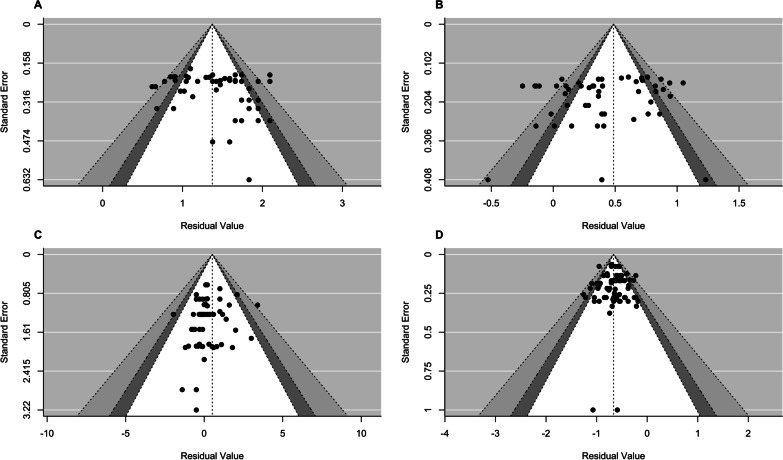
Funnel plots for small-study effects with confidence levels of 90% (white), 95% (dark grey) and 99% (light grey): **A** Intraclass Correlation Coefficient (ICC); **B**: Mean Difference (MD); **C**: Typical Error of measurement (TE); and **(D)**: Correlation Coefficient (*r*). Egger’s regression test: **A** *F* = 0.00, *p* = 0.95; **B** *F* = 0.03, *p* = 0.87; **C** *F* = 0.14, *p* = 0.73; and **D** *F* = 0.01, *p* = 0.91. Begg’s rank correlation test: **A** Kendall's tau = 0.11, *p* = 0.25; **B** Kendall's tau = − 0.05, *p* = 0.54; **C** Kendall’s tau = − 0.10, *p* = 0.29; and **D** Kendall's tau = − 0.05, *p* = 0.57

No indications for small-study effects were observed in the funnel plots (Fig. 7). Begg’s rank correlation and Egger’s regression tests revealed no significant asymmetries for all estimates. Overall, the exclusion of potential outliers in all datasets did not have a practically meaningful influence on the results obtained in the original models (Additional file 7).

## Discussion

In this review, we sought to meta-analyse the measurement properties of SMFT HRex, while additionally examining the modifying effects of athlete and protocol characteristics. Reliability and convergent validity data were obtained from 1218 and 3032 individual observations, drawn from 522 and 1055 team sport athlete inclusions and nested within 29 and 29 independent samples, respectively. We found that the overall TE of SMFT HRex was 1.6% points, with 95% CI ranging from 1.4 to 1.9% points. The width of the 95% PI suggested that future studies may expect to observe TE ranges between 0.9 to 3.0% points. The ICC overall estimate and 95% CI (0.88 [95% CI 0.84 to 0.91]) indicated high to very high relative reliability, and 95% PI ranged from 0.59 to 0.97, implying moderate to very high magnitudes in future studies. We also observed a large inverse relationship (*r* = − 0.58) between SMFT HRex and endurance test results, suggesting its validity as a marker of maximal endurance performance when comparing between athletes. Correlation magnitudes remained consistent across the 95% CI (− 0.54 to − 0.62, large), whereas the interpretation of 95% PI suggested that future studies are likely to observe moderate to very large magnitudes (− 0.31 to − 0.77). Meta-regression analyses suggested that variables related to athlete or protocol characteristics do not have a meaningful effect on these measurement properties.

Our results support previous conclusions [24–26, 34, 74] suggesting no effect of age on the magnitude of SMFT HRex reliability and convergent validity. Although a trend for slightly improved ICC, TE and *r* was observed in older players (Fig. 3–5, panel A and D), the differences were not substantial (Table S2 in Additional file 4). Overall, these results imply that the high-degree of SMFT HRex reliability and convergent validity can be confidently applied among a wide range of age groups, at least bounded by the ages we examined. In this respect, when comparisons were made directly between age extremities of the data (i.e., 10 vs 30 years), there did appear to be meaningful improvements in measurement properties. However, this comparison (e.g., 20 years difference) is likely not relevant in practice, as it spans across a team sport career.

We attempted to enhance the understanding of athlete’s performance status by categorising the studies in reference to their competition level (elite versus sub-elite), although this was only possible for the convergent validity dataset. While the relationship between SMFT HRex and established endurance tests appeared slightly larger in athletes who compete in the highest level of their sport compared to athletes who compete in sub-divisions (Fig. 5, panel F), the difference was negligible (sub-elite to elite: *r* = − 0.56 versus − 0.62, *z*_diff_ = − 0.08 [95% CI − 0.26 to 0.09]). Additionally, while this effect could not be explored in the reliability datasets, the results attained in the individual reliability studies did not indicate any potential trend [25, 28]. These observations would further suggest that athlete characteristics do not appear to meaningfully affect SMFT HRex measurement properties.

We examined the modifying effects of a variety of SMFT characteristics, including the SMFT duration, intensity, and HRex collection method. Collectively, all these modifying effects were trivial or had no clearly meaningful effect. Nonetheless, these findings are practically useful to guide SMFT programming factors in research and practice. Besides being non-exhaustive, another advantage of SMFT is their short duration. The datasets in our meta-analysis included SMFT ranging between 2 and 12 min. Several previous reviews dealing with monitoring HRex [4, 9], together with individual studies investigating the effect of SMFT duration in our meta-analysis [25, 37, 46, 54], have suggested a duration of 3–4 min considered the minimum for HR to attain steady-state during submaximal exercise [4, 75]. In accordance with this physiological rationale, we found that a 4-min SMFT is adequate for obtaining comparable reliability and convergent validity results, with no further meaningful improvements beyond.

Although part of our included studies [25, 46], taken together with previous research in endurance athletes [76, 77], have highlighted an improved absolute reliability (i.e., reduced TE) at higher SMFT intensities, one of the key findings of the current meta-analysis was that SMFT exercise intensity (expressed as HRex) had no effect upon absolute and relative reliability estimates (Figs. 3 and 4, panel B). Indeed, we found somewhat improved estimates of absolute reliability (TE) in lower versus higher intensities, but these differences were clearly trivial (ln_diff_ = 0.06 [95% CI –0.02 to 0.15] for every 5-point % increase in exercise intensity). For example, computing the predicted TE estimates at 80% and 85% HR_max_ indicated 1.48 (95% CI 1.24 to 1.76) and 1.57 (95% CI 1.37 to 1.80) % points, respectively. Likewise, the predicted ICC values for the same intensities were 0.91 (95% CI 0.88 to 0.94) and 0.89 (95% CI 0.86 to 0.92), respectively. Therefore, while marginal differences are evident, we deem them to have no practical substantiality and might therefore be considered trivial.

While higher SMFT HRex values are hypothetically expected to strengthen the relationships with fitness test performance outcomes, because they are collected at intensities closer to exercise cessation, studies examining this effect have reported disparate findings. For example, some of the included studies reported progressive improvements in the relationships with higher SMFT HRex values [35, 74, 78], while others showed the opposite [46, 79] or no differences (comparable correlation magnitudes) [6]. Our meta-regression analysis demonstrated that this effect was mostly compatible with no meaningful change in the magnitude of *r* (*z*_diff_ = − 0.05 [95% CI − 0.16 to 0.05] for every 5-point % increase in HRex; Fig. 5, panel B). To illustrate, predicted estimates for SMFT intensities at 80% and 85% HR_max_ are − 0.52 (95% CI − 0.64 to − 0.38) and − 0.56 (95% CI − 0.62 to − 0.49), respectively, with only the lower CI bounds indicating a possibly small effect. These findings have practical importance and suggest that practitioners do not necessarily require SMFT that elicit higher intensities for adequate HRex reliability and convergent validity. This may be of particular relevance considering SMFT are often administered as part of the warm-up of a training [3, 6, 34, 53], in which an exposure to high intensities maybe considered impractical.

The studies analysed herein used three main collection methods: fixed time point(s) during the test, the mean HR over particular time frames before test cessation (e.g., last 30, 60 s), or mean HR during the overall test (particularly during intermittent variable protocols such as passing drills or small-sided games (SSG); refer to Tables 2 and 3). While using the mean HR during the overall test in intermittent variable (e.g., SSG) SMFT seems a reasonable approach, the rationale for using a particular method and/or time span in other SMFT protocols was not provided in the included studies. While we observed trivial differences between methods (Figs. 3, 4, 5, panel E), mean HR over a particular time-frame before the SMFT cessation maybe deemed preferable [4, 9] since it minimises a potential measurement noise such as HR spikes owing to signal error. In view of the above, it should be noted that in some of the studies [28, 45, 78, 79] which were categorised as ‘fixed’ collection method, HRex was recorded in 2–5 s intervals by default. Moreover, while the collection method used could, in theory, alter HRex values when different intermittent SMFT are administered (see discussion on SMFT category in the following paragraphs), future research may be necessary to elucidate whether different collection approaches should be selected based upon the SMFT category and subsequently the protocol used.

While the present review could not examine the effect of SMFT category, given the small number of independent studies adopting protocols other than those classified as an intermittent incremental, a theoretical discussion is warranted. A fundamental assumption inherent to the use of HRex as a key monitoring measure in SMFT is based on its ability to provide a valid marker of within-athlete relative exercise intensity due to its linear relationship with oxygen uptake at a wide spectrum of submaximal intensities [4, 9]. From a physiological perspective, an essential component of this assumption is that the exercise regimen should be continuous (intends to elicit steady-state) [4, 80]. Interestingly, most of our included studies administered SMFT protocols characterised as intermittent (incremental), including shorter versions of the most commonly used intermittent shuttle fitness tests such as Yo-YoIR1 and 30-15_IFT_. We assume that these particular SMFT were chosen since most of the studies investigated both reliability and convergent validity and thereby match the characteristics of the submaximal and maximal tests or administer them simultaneously. Furthermore, due to the absence of a criterion-standard of evidence-based SMFT protocol(s) [8], it is perhaps more logical to administer shorter versions of established field tests. However, considering the nature of such assessments, which include intermittent running bouts separated by rest periods, the final HRex result is not entirely derived from steady-state exercise and may be influenced by factors related to the reactivation of the cardiac parasympathetic system such as heart rate recovery following each exercise bout. In addition, the multiple CODs required during the test may add a neuromuscular component which may influence the cardiovascular response [75].

To illustrate this concept, one of the most investigated SMFT is the 6-min Yo-YoIR1 [8]. This test requires repeated 2 × 20 m incremental running bouts (shuttles with 180° CODs), interceded by 10 s rest periods [2]. At 6-min of the test, the players are required to run at a mean velocity of 14.5 km h^−1^ (~ 10 s for each running bout and work/rest ratio of 1:1). Assuming that HRex is collected in the last 30 s of the test, the final value includes the mean HRex during 20 s of activity and 10 s of recovery. Another example is the 30-15_IFT_, which includes a fixed work/rest ratio of 2:1, and a longer absolute recovery period between running bouts (15 s) [21]. Assuming a similar volume (6 min) and collection method (mean HRex during the last 30 s), the final HRex value would be derived from 15 s of activity and recovery, respectively. Albeit speculative, this can lead to a less precise and reproducible HRex values considering that heart rate recovery is associated with an inferior degree of reliability [8], and indeed, studies using continuous protocols generally observed better reliability outcomes. Conversely, it could also be argued that intermittent incremental SMFT are preferable given their greater ecological validity to team sports [81] which subsequently can enhance SMFT HRex convergent validity. A primary purpose of future research should therefore include a more explicit examination of continuous versus intermittent SMFT protocols and their link to SMFT HRex measurement properties.

Another aspect of SMFT categories is the use of intermittent variable protocols (SSG or passing drill formats). Despite the relatively large number of the total reliability estimates, only five independent studies met inclusion criteria (four SSG [27, 33, 82, 83] and one passing drill [27]). Collectively, these studies suggested further exploration of such SMFT as they showed comparable absolute and relative reliability values with the values observed in other SMFT generic categories. Nonetheless, considering the range of contextual factors (e.g., technical and tactical elements, exercise structure) that influence locomotor variability [27, 81, 82] and the increased neuromuscular demands [75], caution is necessary in their interpretation. With regards to convergent validity, only two independent studies administering SSG met the inclusion criteria. Interestingly, while one study [82] among senior professional soccer players using 5v5 SSG observed a significant large relationship (*r* = − 0.56) between HRex and the distance covered in Yo-YoIR1, a different study [32] using 3v3 SSG in a similar cohort observed no association (*r* = − 0.22 to − 0.25) with the final velocity achieved in the 30-15_IFT_. Therefore, before using SSG as a standardised SMFT protocol, more research addressing its validity is warranted. Further, to appropriately quantify the influence of locomotor variables upon HRex in the longer-term, we recommend the use of linear regression techniques and avoiding ratios (i.e., SMFT HRex divided by an external intensity parameter) [84].

There are several limitations to our meta-analysis that warrant acknowledgment. First, although we adopted strict inclusion criteria, all of the included studies were observational and conducted with less rigorous methodological design and reporting standards (e.g., STROBE). Second, while it is well known that the main outcome measure (HRex) is influenced by a large number of confounding variables [8] (e.g., environmental conditions, training loads, circadian effect, menstrual cycle stage), only some of the included studies (8 of 30 overall studies; 27%) provided an adequate amount of information regarding how all these confounders were controlled prior and during the test(s) (refer to Fig. 6 ‘confounding variables’ and in Additional file 5: Table S1 for a detailed description). This information is important as such factors may have influenced outcomes of the studies included in our analyses, in particular the reliability findings. For this reason, future studies should report these data descriptively and if needed, account for the factors in the analysis (for example, adjusted HRex based on ‘heat index’ [85]). Third, during the screening process, we identified more potential studies for inclusion. However, since their effect sizes could not be appropriately estimated or were not provided by personal contact, we were not able to include them. With this in mind, we decided to employ a considerably careful evaluation of missing data and selective reporting in the included studies (Fig. 6 and Table S1 in Additional file 5, dimension 5 and 6). As discussed earlier, we also employed different methods to estimate data related to SMFT and/or athlete’s characteristics, all of which could have introduced some errors. Finally, our unbalanced datasets with regard to sex and sport limit the generalization of the findings to female athletes and athletes competing in a particular team sport.

## Practical Implications

We propose several practical implications for researchers and partitioners wishing to use SMFT HRex with a view to determining athlete’s physiological state.

### Protocol Selection

#### Exercise Regimen

While it is as yet unclear whether SMFT protocol category influences different measurement properties of HRex, we recommend the use of continuous protocols until new evidence is available to suggest otherwise. Continuous fixed protocols are likely the easiest to implement into team sport settings, with pitch markings/poles used to demarcate distance and audio cues to direct the running speed (e.g., dictated by a whistle, metronome, pacing tool). When shuttle courses are used, it is suggested to implement longer distances (where applicable) to reduce the number of total CODs and consequently neuromuscular loading.

For example, on a FIFA standard football (soccer) pitch (105 m in length and 68 m in width), performing a 4 min continuous shuttle of pitch width in 20 s repetitions would result in a running speed of 12.68 km h^−1^ and a total number of 12 CODs, assuming the time taken to complete a 180° COD is 0.7 s [86]. Here, it may be pragmatic to marginally adjust the running speed so that the time taken to complete the shuttle is an integer. Another option is to administer a ‘digital figure of eight’ course (e.g., 15 s for 50 m line portions at a mean velocity of 12 km h^−1^), with smaller groups starting at course landmarks. In this approach athletes are exposed to lower neuromuscular loading due to lower COD angles (90° left and right).

#### Exercise Intensity

The current results demonstrated that exercise intensity does not appear to meaningfully affect HRex validity or reliability. Therefore, we recommend the prescription of fixed intensities that serve the training purposes (e.g., integrated into the warm-up and at what stage, start versus end), and must be easily repeated over time (i.e., individual constraints and resources). Running speeds between 10 and 14 km h^−1^ depending on age, level and sport appear reasonable (likely to elicit HRex between ~ 75 and 85% HR_max_ in all team members).

#### Exercise Volume

Protocol durations of 3–4 min have the strongest theoretical and empirical rationale (refer to earlier section discusses SMFT duration in “Discussion” section).

#### HRex Collection Method

During continuous protocols, practitioners are advised to use the mean HR over the last 30–60 s of the test, as athletes have most likely reached a physiological (primarily HR) steady-state and this time window potentially minimises measurement noise related to the wearable device. When using intermittent variable protocols, the mean HRex throughout the overall test is more logical to retain, since exercise intensity is sporadically variable by definition. Regardless of the method used, it is important to visually inspect the HR trace for outliers prior to analysis.

### Conceptual Interpretation of the Data

SMFT HRex can provide a valid marker of endurance performance when comparing between athletes in the same testing bout. That is, an athlete with a lower SMFT HRex might typically be expected to perform better on a standard maximal test on that day versus an athlete with a higher SMFT HRex (and vice versa). This does not necessarily extrapolate to within-athlete comparisons, however, which are usually the focus of training monitoring. Future studies should therefore examine the validity of SMFT HRex to track intra-athlete changes in aerobic capacity.

### Statistical Interpretation of the Data

Under standardised training setting and environmental conditions, SMFT HRex TE can be considered between 1–2% points. The TE can be used to establish thresholds for changes that are beyond the measurement error, which may be useful when interpreting the data to inform decision making regarding an athlete’s physiological state. For example, using our TE pooled estimate of 1.6% points and a *z*-distribution, confidence limits at 80%, 90%, 95% and 99% for an individual change in SMFT HRex are ± 2.9, ± 3.7, ± 4.4 and ± 5.8% points, respectively. These limits are synonymous with the minimum detectable change (MDC), which is the smallest change that can be detected beyond measurement error (which includes normal biological variation in SMFT HRex) [87]. Note that this is not the minimum practically important difference (MPID) or smallest worthwhile change and should not be used as such. The MPID requires separate discussion and can be combined with the MDC to establish both ‘true’ and meaningful changes in SMFT HRex [88].

## Conclusion

**Fig. 8:**
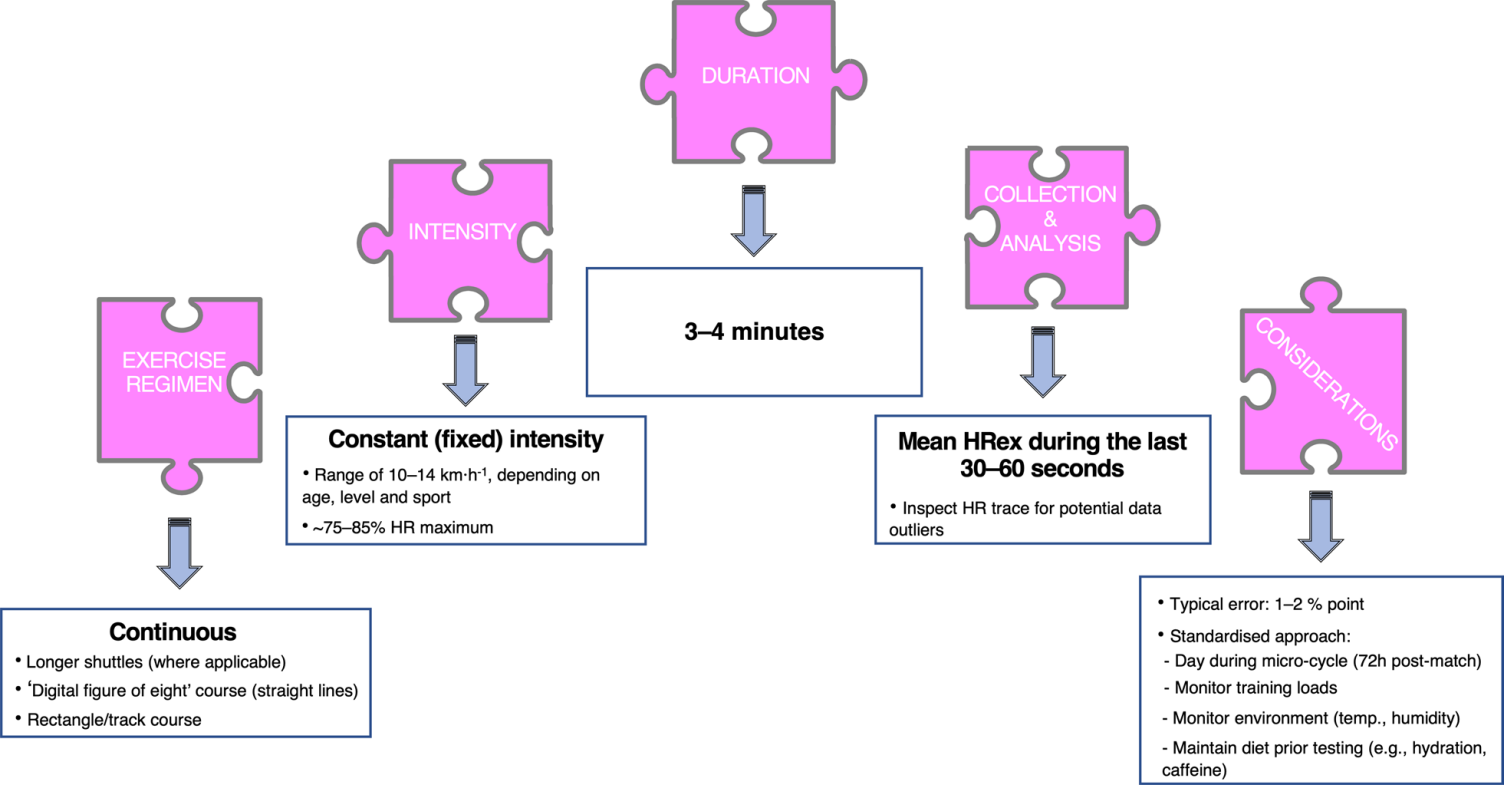
Practical implications for designing and monitoring SMFT HRex in team sports. *temp.* temperature

Our meta-analysis is the first to provide quantitative syntheses of SMFT HRex reliability and convergent validity. Results demonstrate good absolute and high relative reliability, as well as a large convergent validity, which are not affected by athlete or protocol-related characteristics. Practitioners can use these findings to inform SMFT protocol selection (“Practical Implications” section and Fig. 8), interpret data and identify true physiological effects in individual athletes. Future research should focus on SMFT HRex sensitivity to physiological state (within-athlete changes in aerobic capacity), as well as the methodological elements related to SMFT characteristics, HRex collection and analysis approaches that may influence key measurement properties.

## Supplementary Information

**Additional file 1**. Preferred Reporting Items for Systematic Reviews and Meta-Analyses (PRISMA) Checklist.

**Additional file 2**. Methodological overview of the searching strategy, screening process and protocol registration.

**Additional file 3**. Typical error simulation summary and results.

**Additional file 4**. Heterogeneity and meta-regression results from metaanalysis of measurement properties.

**Additional file 5**. A summary detailing the quality assessment criteria of the included studies.

**Additional file 6**. Meta-analysis forest plots of the weighted points estimates.

**Additional file 7**. Sensitivity and influence analyses from metaanalysis of measurement properties.

## Data Availability

Supplementary materials for this paper are also available on the Open Science Framework at https://osf.io/mqnt9/.

## Abbreviations

SMFT: Submaximal fitness tests
HRex: Exercise heart rate
TE: Typical error of measurement
ICC: Intraclass correlation coefficient
MD: Mean difference
*r*: Pearson’s correlation coefficient
Yo-YoIR: Yo-Yo intermittent recovery test
30-15_IFT_: 30-15 Intermittent fitness test
PRISMA: Preferred Reporting Items for Systematic reviews and Meta-Analyses
HR_max_: Heart rate maximum
CV: Coefficient of variation
SD: Standard deviation
CI: Confidence intervals
PI: Prediction intervals
RoBANS: Risk of bias assessment tool for non-randomised studies
COD: Change of direction
MDC: Minimum detectable change
MPID: Minimum practically important difference
