# Supplementary material for: Submaximal Fitness Test in Team Sports: A Systematic Review and Meta-Analysis of Exercise Heart Rate Measurement Properties
Source: Sports Med Open. 2023 Mar 24;9:21. doi: 10.1186/s40798-023-00564-w (PMC10039193; doi:10.1186/s40798-023-00564-w)
Supplement: Supplementary file 1 — Additional file 1. Preferred Reporting Items for Systematic Reviews and Meta-Analyses (PRISMA) Checklist. [file 40798_2023_564_MOESM1_ESM.pdf]

PRISMA 2020 Checklist

Name: Preferred Reporting Items for Systematic Reviews and Meta-Analyses (PRISMA) Checklist

Article Title: Submaximal Fitness Test in Team Sports: A Systematic Review and Meta-Analysis of Exercise Heart Rate Measurement Properties

Journal: Sports Medicine – Open

Authors: Tzlil Shushan[1], Ric Lovell[1,2], Martin Buchheit[3,4,5,6], Tannath J. Scott[7,8], Steve Barrett[9], Dean Norris[1] and Shaun J. McLaren[10,11]

[1] School of Health Sciences, Western Sydney University, Sydney, NSW, Australia

[2] Faculty of Science, Medicine and Health, University of Wollongong, Wollongong, NSW, Australia

[3] HIIT Science, Revelstoke, BC, Canada

[4] French National Institute of Sport (INSEP), Laboratory of Sport, Expertise and Performance (EA 7370), Paris, France

[5] Kitman Labs, Performance Research Intelligence Initiative, Dublin, Ireland

[6] Institute for Health and Sport, Victoria University, Melbourne, VIC, Australia

[7] Netball Australia, Victoria, Australia

[8] Carnegie Applied Rugby Research (CARR) centre, Institute for Sport, Physical Activity and Leisure, Leeds Beckett University, Leeds, UK

[9] Department of Sport Science Innovation, Playermaker, London, United Kingdom

[10] Newcastle Falcons Rugby Club, Newcastle upon Tyne, UK

[11] Institute of sport, Manchester Metropolitan University, Manchester UK

Corresponding Author:

Tzlil Shushan

Email: Tzlil21092@gmail.com

PRISMA 2020 Checklist

Section and Topic	Item #	Checklist item	Location where item is reported
**TITLE**			
Title	1	Proposed title: Submaximal Fitness Test in Team Sports: A Systematic Review and Meta-Analysis of Exercise Heart Rate Measurement Properties	Title
**ABSTRACT**			
Abstract	2	Abstract has met journal criteria instructions. We provided details according to PRISMA abstract checklist (Box 2, items 1–12).	Abstract
**INTRODUCTION**			
Rationale	3	We provided the rationale in the first paragraph of the introduction, listing the importance of reliability and convergent validity measurement properties in sport science.	Introduction, paragraph 2,3,4
Objectives	4	We provided the objectives at the conclusions of the introduction: establish meta-analytic estimate of reliability and convergent validity and examine the influence of putative modifying effects.	Introduction, paragraph 4
**METHODS**			
Eligibility criteria	5	We provided the inclusion criteria in Table 1 and Methods (2.2); "Screening and Study Selection" in the paper	Methods, section 2.2, paragraph 1. Table 1
Information sources	6	We used an extension of the searching strategy and screening process our review paper. The electronic databases MEDLINE, Scopus and Web of Science were used from inception and until January 2022.	Methods, section 2.1, paragraph 1
Search strategy	7	Presented in Methods (2.1); "Registration and Search Strategy" in the paper and more details are provided in Supplementary file S2: 'Methodology Overview'.	Methods, section 2.1, paragraph 1
Selection process	8	We updated the screening process with an extension of the inclusion and exclusion criteria (Table 1 in the paper). The main changes in the inclusion criteria were related to study design; here we accepted studies examining measurement properties of reliability test-retest and correlational designs, and outcome measures; the outcome measure was exercise heart rate only. More details are provided in Supplementary file S2: Methodology Overview	Methods, section 2.2, paragraph 1. Table 1
Data collection process	9	Searching strategy was developed by two authors (TS and RL). Inclusion–exclusion criteria was constructed by three authors (TS, SJM and RL). Data were firstly extracted by the one author (TS) then checked by a second author (SJM). Discrepancies in the extracted were compared and resolved through a discussion (TS, SJM and RL). Discrepancies with the data extracted or missing information were resolved by trying to contact the authors of the study.	Methods, section 2.1–2.4
Data items	10a	We extracted the data as mean and standard deviation of each test-retest (MD), typical error of measurement (TE) and intraclass correlation coefficient (ICC) between test-retest. Person's product-moment correlation coefficient (*r*) indicated convergent validity as the relationship between submaximal exercise heart rate and a criterion measure of a maximal endurance performance. These data include the primary statistical outcomes for the meta-analysis. When data were missing, we used direct contact details of the corresponding authors. Data which could not be obtained either were excluded, obtained from alternative details provided within the text, or omitted from meta-regression analyses.	Methods, section 2.5, paragraphs 1,2,4. Table 2–3
	10b	We extracted data and coded in were 1) the report: author and year of publication; 2) sample characteristics: mean age, age category, sport, gender and competition level; 3) submaximal fitness test characteristics: test category, protocol, duration, intensity; 4) outcome measure: heart rate exercise collection method, 5) endurance performance test and season-phase; and 6) statistical outcomes: methodology used to calculate the typical error and intraclass correlation coefficient. Data which could not be obtained either were excluded, obtained from alternative details	Methods, section 2.3–2.4. Table 2–3. Figure 2

Section and Topic	Item #	Checklist item	Location where item is reported
		provided within the text, or omitted from meta-regression analyses.	
Study risk of bias assessment	11	We assessed the risk of bias of all studies using Risk of Bias Assessment Tool for Non-randomised Studies (RoBANS). RoBANS comprises six-dimension criteria which were answer categories of 'low', 'unclear' and 'high' risk of bias were assigned in each domain. We developed criteria for each domain to answer questions that may influence the reliability or convergent validity results reported in the included studies.	Methods, section 2.7, paragraph 1. Figure 6
Effect measures	12	We included four independent effect estimates: mean difference (MD) and typical error (TE) indicated absolute reliability, intraclass correlation coefficient (ICC) implied relative reliability and Person's product-moment correlation coefficient (*r)* implied convergent validity.	Methods, section 2.5 to 2.6.1, paragraph 1. Table 4
Synthesis methods	13a	The characteristics of the included studies and effect estimates are presented in Table 2–3	Table 2–3
	13b	Data were extracted and tabulated for presentation in the paper and analyses. Missing data that could not obtained either were excluded, obtained from alternative details or estimation strategies presented in Methods (2.4); "Handling Missing Data". If could not obtained data were omitted from meta-regression analyses.	Methods, section 2.4, paragraph 1 to 3
	13c	Results of the overall meta-analysis effect are presented in Table 5 and Results (3.3 and 3.4); "Overall Meta-Analysis" and "Heterogeneity" in the paper. In addition, the Supplementary file S6 includes representation of forest plots. Meta-regression analyses are visualised in Figures 3–5 using bubble plots of the predicted values to the modifying effects.	Results, section 3.3 and 3.4. Table 5. Figure 3–5
	13d	We used multi-level meta-analysis to account for the heterogeneity within-and-between-cluster (i.e., samples). In addition, we conducted Robust Variance Estimation tests to account for the dependency in our data, while accounting for the correlation within samples. We identified statistical heterogeneity using tau and I^2 statistics. All data analyses were conducted using the 'metafor' and 'clubSandwich' packages for R studio environment.	Methods, section 2.6.2 and 2.6.3
	13e	We examined the possible causes of heterogeneity using meta-regression analyses related to SMFT (e.g., exercise intensity) or athletes (e.g., mean age) characteristics. We assessed model strength as the percentage of variance explained by the moderator with Pseudo-R^2.	Methods, section 2.6.3, paragraph 1
	13f	We conducted few methods for sensitivity analysis, including range of correlations between common samples, Cook's distance analysis and Baujat plots to identify potential outliers in Supplementary file S7.	Methods, section 2.8, paragraph 1
Reporting bias assessment	14	We assessed risk of publication bias using the inspection of funnel plots. We also conducted Begg's rank correlation and Egger's regression tests to confirm visual inspection.	Methods, section 2.8, paragraph 1. Figure 7
Certainty assessment	15	We re-ran the models with different correlation magnitudes, omitted potential outliers from the data to examine if the change in the summary estimates and heterogeneity turned substantial.	Methods, section 2.8, paragraph 1
**RESULTS**			
Study selection	16a	Presented in Results (3.1 and 3.2); "Selected Reliability Studies and Characteristics" and "Selected Convergent Validity Studies and Characteristics". PRISMA Flow Chart (Figure 1).	Results, section 3.1 and 3.2,

PRISMA 2020 Checklist

Section and Topic	Item #	Checklist item	Location where item is reported
			Figure 1
	16b	Presented in Methods (2.2); “Screening and Study Selection” and in more details in Supplementary file S2.	Methods, section 2.2, paragraph 1
Study characteristics	17	The characteristics of included studies are presented in Table 2–3 and frequency analysis of these characteristics in Results (3.1 and 3.2); “Selected Reliability Studies and Characteristics” and “Selected Convergent Validity Studies and Characteristics”.	Results, section 3.1–3.2. Table 2–3
Risk of bias in studies	18	Assessment of risk of bias including the sum of each risk category (low, unclear or high) in individual studies and a graphical overview in Figure 6.	Table 2–3. Figure 6
Results of individual studies	19	Forest plots present the results from individual studies 95% confidence, as well as the overall effect, 95% confidence intervals and prediction intervals in Supplementary file S6.	Results, section 3.3, paragraph 1. Table 5.
Results of syntheses	20a	Figure 6 presents the risk of bias across reliability and convergent validity. Supplementary file S5 includes explanation of each domain. Discussion is presented in the limitations within the discussion section.	Discussion, paragraph 11. Figure 6
	20b	The results section includes the statistical syntheses, the summary of estimates and their confidence and prediction intervals. We presented a summary of heterogeneity and uncertainty in the paper and also in Supplementary file S4.	Results, section 3.3 and 3.4
	20c	Meta-regression analyses are presented in Results (3.5); “Meta-Regression Analysis”, Figure 3–5 and Supplementary file S4.	Results, section 3.5, paragraph 1. Figure 3–5
	20d	Cook’s distance summary results and Baujat plots are presented in Supplementary file S7. Sensitivity analyses of different correlations between studies within clusters are in Supplementary file S4.	Results, section 3.6, paragraph 1
Reporting biases	21	Presented in Results (3.6 and 3.7); “Study Qualitative Assessment” and “Small-Study Effects”, and Supplementary file S5 and S7. Explanation of RoBANS and discussion of the limitations in the paper’s Discussion.	Results, section 3.6–3.7. Discussion, paragraph 11
Certainty of evidence	22	Figure 6 presents the risk of bias across reliability and convergent validity and paper’s Discussion. Funnel plot and statistical tests revealed no indication for publication bias. Sensitivity analyses revealed identical results, presented in Supplementary file S7.	Results, section 3.5 to 3.7. Figure 6
**DISCUSSION**			
Discussion	23a	First paragraph in the paper‘s Discussion.	Discussion, paragraph 1

PRISMA 2020 Checklist

Section and Topic	Item #	Checklist item	Location where item is reported
	23b	Discussed within the last paragraph in the paper's Discussion.	Discussion, paragraph 11
	23c	Limitation paragraph in the paper's Discussion and Supplementary file S2.	Discussion, paragraph 11
	23d	Last paragraph in the paper's Discussion, practical implications and conclusions in the paper.	Discussion, section 4, paragraph 1–10. Practical Implications, section 5.1–5.2. Figure 8
**OTHER INFORMATION**			
Registration and protocol	24a	Our systematic review and meta-analysis was registered in Open Science Framework (OSF) on the 8th of September (DOI: https://doi.org/10.17605/OSF.IO/9C2JV). The Registration was conducted while having the data (due to the nature of the extended inclusion criteria), however before to data analyses. Explanation is also presented in Supplementary file S2.	Declarations, registration
	24b	Review protocols can be accessed at DOI: https://doi.org/10.17605/OSF.IO/9C2JV.	Declarations, registration
	24c	No amendments to information provided at registration were made.	NA
Support	25	Reported in accordance with the journal guidelines. No sources of financial or non-financial support for the review, and no role of the funders or sponsors in the review.	Declarations, funding
Competing interests	26	Any competing interests of review authors declared (none).	Declarations, competing interest
Availability of data, code and other materials	27	Report which of the following are publicly available and where they can be found in the Open Science Framework by accessing: https://osf.io/mqnt9/.	Declarations, availability of data and material

From: Page MJ, McKenzie JE, Bossuyt PM, Boutron I, Hoffmann TC, Mulrow CD, et al. The PRISMA 2020 statement: an updated guideline for reporting systematic reviews. BMJ 2021;372:n71. doi: 10.1136/bmj.n71

For more information, visit: http://www.prisma-statement.org/

PRISMA 2020 Checklist

Section and Topic	Item #	Checklist item	Location where item is reported
**TITLE**			
Title	1	Identify the report as a systematic review.	
**ABSTRACT**			
Abstract	2	See the PRISMA 2020 for Abstracts checklist.	
**INTRODUCTION**			
Rationale	3	Describe the rationale for the review in the context of existing knowledge.	
Objectives	4	Provide an explicit statement of the objective(s) or question(s) the review addresses.	
**METHODS**			
Eligibility criteria	5	Specify the inclusion and exclusion criteria for the review and how studies were grouped for the syntheses.	
Information sources	6	Specify all databases, registers, websites, organisations, reference lists and other sources searched or consulted to identify studies. Specify the date when each source was last searched or consulted.	
Search strategy	7	Present the full search strategies for all databases, registers and websites, including any filters and limits used.	
Selection process	8	Specify the methods used to decide whether a study met the inclusion criteria of the review, including how many reviewers screened each record and each report retrieved, whether they worked independently, and if applicable, details of automation tools used in the process.	
Data collection process	9	Specify the methods used to collect data from reports, including how many reviewers collected data from each report, whether they worked independently, any processes for obtaining or confirming data from study investigators, and if applicable, details of automation tools used in the process.	
Data items	10a	List and define all outcomes for which data were sought. Specify whether all results that were compatible with each outcome domain in each study were sought (e.g. for all measures, time points, analyses), and if not, the methods used to decide which results to collect.	
	10b	List and define all other variables for which data were sought (e.g. participant and intervention characteristics, funding sources). Describe any assumptions made about any missing or unclear information.	
Study risk of bias assessment	11	Specify the methods used to assess risk of bias in the included studies, including details of the tool(s) used, how many reviewers assessed each study and whether they worked independently, and if applicable, details of automation tools used in the process.	
Effect measures	12	Specify for each outcome the effect measure(s) (e.g. risk ratio, mean difference) used in the synthesis or presentation of results.	
Synthesis methods	13a	Describe the processes used to decide which studies were eligible for each synthesis (e.g. tabulating the study intervention characteristics and comparing against the planned groups for each synthesis (item #5)).	
	13b	Describe any methods required to prepare the data for presentation or synthesis, such as handling of missing summary statistics, or data conversions.	
	13c	Describe any methods used to tabulate or visually display results of individual studies and syntheses.	
	13d	Describe any methods used to synthesize results and provide a rationale for the choice(s). If meta-analysis was performed, describe the model(s), method(s) to identify the presence and extent of statistical heterogeneity, and software package(s) used.	
	13e	Describe any methods used to explore possible causes of heterogeneity among study results (e.g. subgroup analysis, meta-regression).	
	13f	Describe any sensitivity analyses conducted to assess robustness of the synthesized results.	
Reporting bias assessment	14	Describe any methods used to assess risk of bias due to missing results in a synthesis (arising from reporting biases).	
Certainty assessment	15	Describe any methods used to assess certainty (or confidence) in the body of evidence for an outcome.	

PRISMA 2020 Checklist

Section and Topic	Item #	Checklist item	Location where item is reported
**RESULTS**			
Study selection	16a	Describe the results of the search and selection process, from the number of records identified in the search to the number of studies included in the review, ideally using a flow diagram.	
	16b	Cite studies that might appear to meet the inclusion criteria, but which were excluded, and explain why they were excluded.	
Study characteristics	17	Cite each included study and present its characteristics.	
Risk of bias in studies	18	Present assessments of risk of bias for each included study.	
Results of individual studies	19	For all outcomes, present, for each study: (a) summary statistics for each group (where appropriate) and (b) an effect estimate and its precision (e.g. confidence/credible interval), ideally using structured tables or plots.	
Results of syntheses	20a	For each synthesis, briefly summarise the characteristics and risk of bias among contributing studies.	
	20b	Present results of all statistical syntheses conducted. If meta-analysis was done, present for each the summary estimate and its precision (e.g. confidence/credible interval) and measures of statistical heterogeneity. If comparing groups, describe the direction of the effect.	
	20c	Present results of all investigations of possible causes of heterogeneity among study results.	
	20d	Present results of all sensitivity analyses conducted to assess the robustness of the synthesized results.	
Reporting biases	21	Present assessments of risk of bias due to missing results (arising from reporting biases) for each synthesis assessed.	
Certainty of evidence	22	Present assessments of certainty (or confidence) in the body of evidence for each outcome assessed.	
**DISCUSSION**			
Discussion	23a	Provide a general interpretation of the results in the context of other evidence.	
	23b	Discuss any limitations of the evidence included in the review.	
	23c	Discuss any limitations of the review processes used.	
	23d	Discuss implications of the results for practice, policy, and future research.	
**OTHER INFORMATION**			
Registration and protocol	24a	Provide registration information for the review, including register name and registration number, or state that the review was not registered.	
	24b	Indicate where the review protocol can be accessed, or state that a protocol was not prepared.	
	24c	Describe and explain any amendments to information provided at registration or in the protocol.	
Support	25	Describe sources of financial or non-financial support for the review, and the role of the funders or sponsors in the review.	
Competing interests	26	Declare any competing interests of review authors.	
Availability of data, code and other materials	27	Report which of the following are publicly available and where they can be found: template data collection forms; data extracted from included studies; data used for all analyses; analytic code; any other materials used in the review.	
