# Supplementary material for: Submaximal Fitness Test in Team Sports: A Systematic Review and Meta-Analysis of Exercise Heart Rate Measurement Properties
Source: Sports Med Open. 2023 Mar 24;9:21. doi: 10.1186/s40798-023-00564-w (PMC10039193; doi:10.1186/s40798-023-00564-w)
Supplement: Supplementary file 2 — Additional file 2. Methodological overview of the searching strategy, screening process and protocol registration. [file 40798_2023_564_MOESM2_ESM.pdf]

Name: Methodological overview of the searching strategy, screening process and protocol registration

Article Title: Submaximal Fitness Test in Team Sports: A Systematic Review and Meta-Analysis of Exercise Heart Rate Measurement Properties

Journal: Sports Medicine – Open

Authors: Tzlil Shushan[1], Ric Lovell[1,2], Martin Buchheit[3,4,5,6], Tannath J. Scott[7,8], Steve Barrett[9], Dean Norris[1] and Shaun J. McLaren[10,11]

[1] School of Health Sciences, Western Sydney University, Sydney, NSW, Australia

[2] Faculty of Science, Medicine and Health, University of Wollongong, Wollongong, NSW, Australia

[3] HIIT Science, Revelstoke, BC, Canada

[4] French National Institute of Sport (INSEP), Laboratory of Sport, Expertise and Performance (EA 7370), Paris, France

[5] Kitman Labs, Performance Research Intelligence Initiative, Dublin, Ireland

[6] Institute for Health and Sport, Victoria University, Melbourne, VIC, Australia

[7] Netball Australia, Victoria, Australia

[8] Carnegie Applied Rugby Research (CARR) centre, Institute for Sport, Physical Activity and Leisure, Leeds Beckett University, Leeds, UK

[9] Department of Sport Science Innovation, Playermaker, London, United Kingdom

[10] Newcastle Falcons Rugby Club, Newcastle upon Tyne, UK

[11] Institute of sport, Manchester Metropolitan University, Manchester UK

Corresponding Author:

Tzlil Shushan

Email: Tzlil21092@gmail.com

Background for the Current Meta-Analysis

In our original review we aim to conduct a comprehensive systematic review and narrative synthesis on the utility of Submaximal Fitness Tests (SMFT) in team-sports. In accordance with our definition and characteristics of SMFT (see our previous review https://doi.org/10.1007/s40279-022-01712-0, section 3.1 'Submaximal Fitness Tests Definition' and Fig. 2 in the current paper), we accepted all studies (n = 87) administering SMFT in a) test-retest or correlational study designs; b) observational with single/repeated measures designs with or without reporting training loads, training context or environment; and c) training intervention studies that incorporated pre and post measurements. Furthermore, we sought to synthesise the current knowledge on all possible outcome measures, including cardiorespiratory/metabolic, subjective or mechanical.

We found a considerable amount of studies examining the reliability and convergent validity of SMFT exercise heart rate (HRex) in team-sport athletes. Since HRex has become the most common outcome measure in SMFT and is considered to have high feasibility in team sports, improved understanding on its measurement properties can assist practitioners to have a better inference approach regarding athlete's current physiological state and training effects.

Screening and Study Selection

As shown in paper (Fig. 1, PRISMA Flow Chart), for the current meta-analysis we updated the screening process with an extension of the inclusion and exclusion criteria (Table 1 in the paper). The main changes in the inclusion criteria were related to study design; here we accepted studies examining measurement properties of reliability test-retest and correlational designs, and outcome measures; the outcome measure was exercise heart rate only. From the original 87 studies included in our original review, 52 studies were excluded based on their study design (Table 1, criterion 5 in the paper), 4 studies did not use exercise heart rate as outcome measure (criterion 8), and in 2 studies statistical estimates could not be obtained (criterion 5), either from personal contact of the authors, or estimation strategies. We retrieved one more study from updated searches. The final data included 30 overall independent studies, with 21 (29 unique samples) and 20 (29 unique samples) providing reliability and convergent validity data, respectively.

Registration

This systemic review and meta-analysis was registered in Open Science Framework (available through https://doi.org/10.17605/OSF.IO/9C2JV). Whilst the registration was conducted after having access to most of the data and conducting some explanatory analysis presented in the previous review, given that this is a follow up research project, we still sought that a separate registration should be documented.

Searching Strategy Syntaxes

MEDLINE (Strategy 1)

1 submaximal.mp. [mp=title, abstract, original title, name of substance word, subject heading word, floating sub-heading word, keyword heading word, organism supplementary concept word, protocol supplementary concept word, rare disease supplementary concept word, unique identifier, synonyms] (14701)
2 sub-maximal.mp. [mp=title, abstract, original title, name of substance word, subject heading word, floating sub-heading word, keyword heading word, organism supplementary concept word, protocol supplementary concept word, rare disease supplementary concept word, unique identifier, synonyms] (1223)
3 "sub maximal".mp. [mp=title, abstract, original title, name of substance word, subject heading word, floating sub-heading word, keyword heading word, organism supplementary concept word, protocol supplementary concept word, rare disease supplementary concept word, unique identifier, synonyms] (1223)
4 standardised.mp. [mp=title, abstract, original title, name of substance word, subject heading word, floating sub-heading word, keyword heading word, organism supplementary concept word, protocol supplementary concept word, rare disease supplementary concept word, unique identifier, synonyms] (33106)
5 standardized.mp. [mp=title, abstract, original title, name of substance word, subject heading word, floating sub-heading word, keyword heading word, organism supplementary concept word, protocol supplementary concept word, rare disease supplementary concept word, unique identifier, synonyms] (198666)
6 1 or 2 or 3 or 4 or 5 (246752)
7 exercise.mp. [mp=title, abstract, original title, name of substance word, subject heading word, floating sub-heading word, keyword heading word, organism supplementary concept word, protocol supplementary concept word, rare disease supplementary concept word, unique identifier, synonyms] (340290)
8 test.mp. [mp=title, abstract, original title, name of substance word, subject heading word, floating sub-heading word, keyword heading word, organism supplementary concept word, protocol supplementary concept word, rare disease supplementary concept word, unique identifier, synonyms] (1551077)
9 drill.mp. [mp=title, abstract, original title, name of substance word, subject heading word, floating sub-heading word, keyword heading word, organism supplementary concept word, protocol supplementary concept word, rare disease supplementary concept word, unique identifier, synonyms] (6662)
10 7 or 8 or 9 (1797753)
11 fitness.mp. [mp=title, abstract, original title, name of substance word, subject heading word, floating sub-heading word, keyword heading word, organism supplementary concept word, protocol supplementary concept word, rare disease supplementary concept word, unique identifier, synonyms] (87734)
12 fatigue.mp. [mp=title, abstract, original title, name of substance word, subject heading word, floating sub-heading word, keyword heading word, organism supplementary concept word, protocol supplementary concept word, rare disease supplementary concept word, unique identifier, synonyms] (103599)
13 11 or 12 (189408)
14 athletes.mp. [mp=title, abstract, original title, name of substance word, subject heading word, floating sub-heading word, keyword heading word, organism supplementary concept word, protocol supplementary concept word, rare disease supplementary concept word, unique identifier, synonyms] (48284)
15 players.mp. [mp=title, abstract, original title, name of substance word, subject heading word, floating sub-heading word, keyword heading word, organism supplementary concept word, protocol supplementary concept word, rare disease supplementary concept word, unique identifier, synonyms] (39760)
16 "team sport".mp. [mp=title, abstract, original title, name of substance word, subject heading word, floating sub-heading word, keyword heading word, organism supplementary concept word, protocol supplementary concept word, rare disease supplementary concept word, unique identifier, synonyms] (1082)
17 "team sports".mp. [mp=title, abstract, original title, name of substance word, subject heading word, floating sub-heading word, keyword heading word, organism supplementary concept word, protocol supplementary concept word, rare disease supplementary concept word, unique identifier, synonyms] (1346)
18 "team based sport".mp. [mp=title, abstract, original title, name of substance word, subject heading word, floating sub-heading word, keyword heading word, organism supplementary concept word, protocol supplementary concept word, rare disease supplementary concept word, unique identifier, synonyms] (7)
19 14 or 15 or 16 or 17 or 18 (81830)
20 6 and 10 and 13 and 19 (375)

MEDLINE (Strategy 2)

1 submaximal.mp. [mp=title, abstract, original title, name of substance word, subject heading word, floating sub-heading word, keyword heading word, organism supplementary concept word, protocol supplementary concept word, rare disease supplementary concept word, unique identifier, synonyms] (14701)
2 sub-maximal.mp. [mp=title, abstract, original title, name of substance word, subject heading word, floating sub-heading word, keyword heading word, organism supplementary concept word, protocol supplementary concept word, rare disease supplementary concept word, unique identifier, synonyms] (1223)
3 "sub maximal".mp. [mp=title, abstract, original title, name of substance word, subject heading word, floating sub-heading word, keyword heading word, organism supplementary concept word, protocol supplementary concept word, rare disease supplementary concept word, unique identifier, synonyms] (1223)
4 standardised.mp. [mp=title, abstract, original title, name of substance word, subject heading word, floating sub-heading word, keyword heading word, organism supplementary concept word, protocol supplementary concept word, rare disease supplementary concept word, unique identifier, synonyms] (33106)
5 standardized.mp. [mp=title, abstract, original title, name of substance word, subject heading word, floating sub-heading word, keyword heading word, organism supplementary concept word, protocol supplementary concept word, rare disease supplementary concept word, unique identifier, synonyms] (198666)
6 1 or 2 or 3 or 4 or 5 (246752)
7 exercise.mp. [mp=title, abstract, original title, name of substance word, subject heading word, floating sub-heading word, keyword heading word, organism supplementary concept word, protocol supplementary concept word, rare disease supplementary concept word, unique identifier, synonyms] (340290)
8 test.mp. [mp=title, abstract, original title, name of substance word, subject heading word, floating sub-heading word, keyword heading word, organism supplementary concept word, protocol supplementary concept word, rare disease supplementary concept word, unique identifier, synonyms] (1551077)

9 drill.mp. [mp=title, abstract, original title, name of substance word, subject heading word, floating sub-heading word, keyword heading word, organism supplementary concept word, protocol supplementary concept word, rare disease supplementary concept word, unique identifier, synonyms] (6662)
10 7 or 8 or 9 (1797753)
11 physiological.mp. [mp=title, abstract, original title, name of substance word, subject heading word, floating sub-heading word, keyword heading word, organism supplementary concept word, protocol supplementary concept word, rare disease supplementary concept word, unique identifier, synonyms] (763561)
12 metabolic.mp. [mp=title, abstract, original title, name of substance word, subject heading word, floating sub-heading word, keyword heading word, organism supplementary concept word, protocol supplementary concept word, rare disease supplementary concept word, unique identifier, synonyms] (549587)
13 psychological.mp. [mp=title, abstract, original title, name of substance word, subject heading word, floating sub-heading word, keyword heading word, organism supplementary concept word, protocol supplementary concept word, rare disease supplementary concept word, unique identifier, synonyms] (473066)
14 psychometric.mp. [mp=title, abstract, original title, name of substance word, subject heading word, floating sub-heading word, keyword heading word, organism supplementary concept word, protocol supplementary concept word, rare disease supplementary concept word, unique identifier, synonyms] (41658)
15 mechanical.mp. [mp=title, abstract, original title, name of substance word, subject heading word, floating sub-heading word, keyword heading word, organism supplementary concept word, protocol supplementary concept word, rare disease supplementary concept word, unique identifier, synonyms] (378553)
16 biomechanical.mp. [mp=title, abstract, original title, name of substance word, subject heading word, floating sub-heading word, keyword heading word, organism supplementary concept word, protocol supplementary concept word, rare disease supplementary concept word, unique identifier, synonyms] (136147)
17 11 or 12 or 13 or 14 or 15 or 16 (2183069)
18 response.mp. [mp=title, abstract, original title, name of substance word, subject heading word, floating sub-heading word, keyword heading word, organism supplementary concept word, protocol supplementary concept word, rare disease supplementary concept word, unique identifier, synonyms] (2391179)
19 responses.mp. [mp=title, abstract, original title, name of substance word, subject heading word, floating sub-heading word, keyword heading word, organism supplementary concept word, protocol supplementary concept word, rare disease supplementary concept word, unique identifier, synonyms] (898430)
20 18 or 19 (2905304)
21 athletes.mp. [mp=title, abstract, original title, name of substance word, subject heading word, floating sub-heading word, keyword heading word, organism supplementary concept word, protocol supplementary concept word, rare disease supplementary concept word, unique identifier, synonyms] (48284)
22 players.mp. [mp=title, abstract, original title, name of substance word, subject heading word, floating sub-heading word, keyword heading word, organism supplementary concept word, protocol supplementary concept word, rare disease supplementary concept word, unique identifier, synonyms] (39760)
23 "team sport".mp. [mp=title, abstract, original title, name of substance word, subject heading word, floating sub-heading word, keyword heading word, organism supplementary concept word, protocol supplementary concept word, rare disease supplementary concept word, unique identifier, synonyms] (1082)
24 "team sports".mp. [mp=title, abstract, original title, name of substance word, subject heading word, floating sub-heading word, keyword heading word, organism supplementary concept word, protocol supplementary concept word, rare disease supplementary concept word, unique identifier, synonyms] (1346)
25 "team based sport".mp. [mp=title, abstract, original title, name of substance word, subject heading word, floating sub-heading word, keyword heading word, organism supplementary concept word, protocol supplementary concept word, rare disease supplementary concept word, unique identifier, synonyms] (7)
26 21 or 22 or 23 or 24 or 25 (81830)
27 6 and 10 and 17 and 20 and 26 (199)

Web of Science (Strategy 1)
TOPIC: (Submaximal OR sub - maximal OR "sub maximal" OR standardised OR standardized) AND TOPIC: (exercise OR test OR drill) AND TOPIC: (fitness OR fatigue) AND TOPIC: ("athletes" OR "players" OR "team sport" OR "team sports" OR "team based sport")

Web of Science (Strategy 2)
TOPIC: (Submaximal OR sub - maximal OR "sub maximal" OR standardised OR standardized) AND TOPIC: (exercise OR test OR drill) AND TOPIC: (physiological OR metabolic OR psychological OR psychometric OR mechanical OR biomechanical) AND TOPIC: (response OR responses) AND TOPIC: (athletes OR players OR "team sport" OR "team sports" OR "team based sport")

Scopus (Strategy 1)
(TITLE-ABS-KEY (submaximal OR sub-maximal OR "sub maximal" OR standardised OR standardized) AND TITLE-ABS-KEY (exercise OR test OR drill) AND TITLE-ABS-KEY (fitness OR fatigue) AND TITLE-ABS-KEY (athletes OR players OR "team sport" OR "team sports" OR "team based sport"))

Scopus (Strategy 2)
(TITLE-ABS-KEY (submaximal OR sub-maximal OR "sub maximal" OR standardised OR standardized) AND TITLE-ABS-KEY (exercise OR test OR drill) AND TITLE-ABS-KEY (physiological OR metabolic OR psychological OR psychometric OR mechanical OR biomechanical) AND TITLE-ABS-KEY (response OR responses) AND TITLE-ABS-KEY (athletes OR players OR "team sport" OR "team sports" OR "team based sport"))
