# Supplementary material for: Submaximal Fitness Test in Team Sports: A Systematic Review and Meta-Analysis of Exercise Heart Rate Measurement Properties
Source: Sports Med Open. 2023 Mar 24;9:21. doi: 10.1186/s40798-023-00564-w (PMC10039193; doi:10.1186/s40798-023-00564-w)
Supplement: Supplementary file 4 — Additional file 4. Heterogeneity and meta-regression results from metaanalysis of measurement properties. [file 40798_2023_564_MOESM4_ESM.pdf]

Name: Heterogeneity and meta-regression results from meta-analysis of measurement properties

Article Title: Submaximal Fitness Test in Team Sports: A Systematic Review and Meta-Analysis of Exercise Heart Rate Measurement Properties

Journal: Sports Medicine – Open

Authors: Tzlil Shushan[1], Ric Lovell[1,2], Martin Buchheit[3,4,5,6], Tannath J. Scott[7,8], Steve Barrett[9], Dean Norris[1] and Shaun J. McLaren[10,11]

[1] School of Health Sciences, Western Sydney University, Sydney, NSW, Australia

[2] Faculty of Science, Medicine and Health, University of Wollongong, Wollongong, NSW, Australia

[3] HIIT Science, Revelstoke, BC, Canada

[4] French National Institute of Sport (INSEP), Laboratory of Sport, Expertise and Performance (EA 7370), Paris, France

[5] Kitman Labs, Performance Research Intelligence Initiative, Dublin, Ireland

[6] Institute for Health and Sport, Victoria University, Melbourne, VIC, Australia

[7] Netball Australia, Victoria, Australia

[8] Carnegie Applied Rugby Research (CARR) centre, Institute for Sport, Physical Activity and Leisure, Leeds Beckett University, Leeds, UK

[9] Department of Sport Science Innovation, Playermaker, London, United Kingdom

[10] Newcastle Falcons Rugby Club, Newcastle upon Tyne, UK

[11] Institute of sport, Manchester Metropolitan University, Manchester UK

Corresponding Author:

Tzlil Shushan

Email: Tzlil21092@gmail.com

Table S1 Heterogeneity statistics from meta-analysis of measurement properties

**Measurement Property**	**Statistic**	***k***	***n***	**I^2_2 (95% CI)**	**I^2_3 (95% CI)**	**τ_2 (95% CI)**	**τ_3 (95% CI)**
Reliability	MD	24	68	0.5% (0.0% to 14.2%)	0.0% (0.0% to 26.4%)	0.09 (0.00 to 0.49)	0.00 (0.00 to 0.72)
	TE	24	51	43.4% (42.8% to 76.8%)	28.4% (0.0% to 78.3%)	0.23 (0.15 to 0.32)	0.18 (0.00 to 0.34)
	ICC	26	53	39.4% (35.0% to 68.3%)	24.4% (0.0% to 72.5%)	0.26 (0.18 to 0.36)	0.20 (0.00 to 0.40)
Convergent Validity	*r*	28	72	48.3% (33.0% to 62.9%)	0.0% (0.0% to 42.6%)	0.16 (0.11 to 0.21)	0.00 (0.00 to 0.14)

ICC intraclass correlation; *k* number of studies; *MD* mean difference; *n* number of estimates; *r* correlation coefficient; *TE* typical error; τ_2 tau level 2; τ_3 tau level 3; I^2_2 I^2 level 2; I^2_3 I^2 level 3

Table S2 Meta-regression of measurement properties

Modifying Effect	*k*	n		Intercept (95%CI)<br>Slope (95%CI)	Predicted Estimate (95%CI)	Interpretation of the Effect	R^2_2	R^2_3	p_{MET}
ICC									
Mean age	26	53	18y	1.37 (1.21 to 1.53)	0.88 (0.83 to 0.91)	trivial	0%	11%	0.21
			+3y	0.06 (–0.04 to 0.15)	0.89 (0.84 to 0.92)				
Exercise intensity HRex	25	49	80%	1.53 (1.35 to 1.71)	0.91 (0.88 to 0.94)	trivial	0%	52%	0.03
			+5%	–0.1 (–0.20 to –0.01)	0.89 (0.86 to 0.92)				
Duration	27	54	4min	1.41 (1.22 to 1.61)	0.89 (0.83 to 0.93)	trivial	0%	0%	0.16
			+2min	–0.04 (–0.11 to 0.03)	0.88 (0.83 to (0.92)				
Age category	27	54	Youth	1.36 (1.14 to 1.58)	0.88 (0.81 to 0.92)	trivial	0%	0%	0.87
			Senior	0.02 (–0.29 to 0.34)	0.88 (0.81 to 0.93)				
HR collection method	27	54	Fixed	1.33 (1.07 to 1.58)	0.87 (0.79 to 0.92)	trivial	1%	0%	
			Mean R	0.16 (–0.21 to 0.54)	0.90 (0.82 to 0.95)				0.37
			Mean O	–0.09 (–0.50 to 0.32)	0.84 (0.72 to 0.92)				0.64
TE									
Mean age	25	52	18y	0.50 (0.38 to 0.62)	1.65 (1.43 to 1.89)	trivial	2%	45%	0.04
			+3y	–0.07 (–0.14 to –0.0001)	1.53 (1.34 to 1.75)				
Exercise intensity HRex	25	52	80%	0.39 (0.21 to 0.57)	1.48 (1.24 to 1.76)	trivial	0%	30%	0.13
			+5%	0.06 (–0.02 to 0.15)	1.57 (1.37 to 1.80)				
Duration	25	52	4min	0.49 (0.34 to 0.64)	1.61 (1.20 to 2.17)	trivial	0%	0%	0.93
			+2min	–0.003 (–0.08 to –0.08)	1.60 (1.04 to 2.48)				
Age category	25	52	Youth	0.58 (0.42 to 0.75)	1.79 (1.52 to 2.11)	mostly trivial	6%	25%	0.08
			Senior	–0.22 (–0.47 to 0.03)	1.44 (1.18 to 1.76)				
HR collection method	25	52	Fixed	0.64 (0.43 to 0.85)	1.90 (1.54 to 2.33)	mostly trivial	0%	28%	
			Mean R	–0.22 (–0.50 to 0.05)	1.52 (1.21 to 1.92)				0.11
			Mean O	–0.27 (–0.64 to 0.10)	1.45 (1.03 to 2.03)				0.14
r									
Mean age	28	71	18y	–0.63 (–0.71 to –0.55)	–0.56 (–0.61 to –0.50)	trivial	2%		0.42
			+3y	–0.02 (–0.07 to 0.03)	–0.57 (–0.61 to –0.53)				
Exercise intensity HRex	28	64	80%	–0.58 (–0.75 to –0.40)	–0.52 (–0.64 to –0.38)	mostly trivial	12%		0.28
			+5%	–0.05 (–0.16 to 0.05)	–0.56 (–0.62 to –0.49)				
Duration	29	73	4min	–0.62 (–0.71 to -0.53)	–0.55 (–0.62 to –0.48)	trivial	4%		0.20

			+2	–0.08 (–0.21 to 0.05)	–0.60 (–0.66 to –0.54)			
Age category	29	73	Youth	–0.65 (–0.78 to –0.51)	–0.57 (–0.65 to –0.47)	trivial	0%	0.71
			Senior	–0.03 (–0.17 to –0.12)	–0.59 (–0.64 to –0.52)			
Level	23	60	Non-Elite	–0.64 (–0.79 to –0.49)	–0.56 (–0.66 to –0.45)	trivial	5%	0.30
			Elite	–0.08 (–0.26 to 0.09)	–0.62 (–0.73 to –0.48)			
HR collection method	27	69	Fixed	–0.70 (–0.79; –0.62)	–0.61 (–0.66 to –0.55)	trivial	0%	0.27
			Mean R	0.10 (–0.09 to 0.29)	–0.54 (–0.65 to –0.41)			

CI confidence intervals; *ICC* intraclass correlation; *ISRT* interval shuttle run test; *k* number of studies; *min* minute; *MD* mean difference; *Mean O* mean overall*, Mean R* mean range, *n* number of estimates; p_{MET} *P* values; *r* correlation coefficient; $R^2_{[2]}$ pseudo-R^2 level 2; $R^2_{[3]}$ pseudo-R^2 level 3; *TE* typical error; *Yo-YoIR1* yo-yo intermittent recovery test level 1; *y* years; *Yo-YoIR2* yo-yo intermittent recovery test level 2; ICC and correlation r coefficients are presented in fisher's z values. TE coefficients are presented in log-transformed values. The predicted estimates are presented in the back-transformed original values.

Table S3 Sensitivity analysis

**Measurement property**	*ρ*	***Estimate***	***95% CI (lower; upper)***	τ^2_2	τ^2_3
ICC	.0	0.88	0.84; 0.91	0.17	0.28
	.1	0.88	0.84; 0.91	0.20	0.26
	.3	0.88	0.84; 0.91	0.23	0.24
	.5	0.88	0.84; 0.91	0.25	0.21
	.7	0.88	0.84; 0.91	0.27	0.19
MD	.0	0.27	–0.15; 0.63	0.00	0.15
	.1	0.38	0.01; 0.74	0.00	0.01
	.3	0.46	0.08; 0.83	0.00	0.00
	.5	0.50	0.13; 0.7	0.00	0.00
	.7	0.53	0.16; 0.9	0.28	0.01
TE	.0	1.6	1.4; 1.8	0.20	0.20
	.01	1.6	1.4; 1.9	0.21	0.21
	.09	1.6	1.4; 1.9	0.21	0.21
	.25	1.6	1.4; 1.9	0.22	0.19
	.5	1.6	1.4; 1.9	0.24	0.17
r	.0	–0.58	–0.62; –0.53	0.12	0.05
	.1	–0.58	–0.62; –0.53	0.12	0.00
	.3	–0.58	–0.62; –0.54	0.13	0.00
	.5	–0.58	–0.62; –0.54	0.15	0.00
	.7	–0.59	–0.63; –0.54	0.17	0.00

CI confidence intervals; *ICC* intraclass correlation; *MD* mean difference; *ρ* correlation; $\tau_{[2]}$ tau level 2; $\tau_{[3]}$ tau level 3; *r* correlation coefficient; *TE* typical error. *ρ*=.0 implies using sampling variance. TE *ρ* are slightly different due to the structure of the covariance formula of ln SD.
