# Supplementary material for: Submaximal Fitness Test in Team Sports: A Systematic Review and Meta-Analysis of Exercise Heart Rate Measurement Properties
Source: Sports Med Open. 2023 Mar 24;9:21. doi: 10.1186/s40798-023-00564-w (PMC10039193; doi:10.1186/s40798-023-00564-w)
Supplement: Supplementary file 5 — Additional file 5. A summary detailing the quality assessment criteria of the included studies. [file 40798_2023_564_MOESM5_ESM.pdf]

Name: A summary detailing the quality assessment criteria of the included studies

Article Title: Submaximal Fitness Test in Team Sports: A Systematic Review and Meta-Analysis of Exercise Heart Rate Measurement Properties

Journal: Sports Medicine – Open

Authors: Tzlil Shushan[1], Ric Lovell[1,2], Martin Buchheit[3,4,5,6], Tannath J. Scott[7,8], Steve Barrett[9], Dean Norris[1] and Shaun J. McLaren[10,11]

[1] School of Health Sciences, Western Sydney University, Sydney, NSW, Australia

[2] Faculty of Science, Medicine and Health, University of Wollongong, Wollongong, NSW, Australia

[3] HIIT Science, Revelstoke, BC, Canada

[4] French National Institute of Sport (INSEP), Laboratory of Sport, Expertise and Performance (EA 7370), Paris, France

[5] Kitman Labs, Performance Research Intelligence Initiative, Dublin, Ireland

[6] Institute for Health and Sport, Victoria University, Melbourne, VIC, Australia

[7] Netball Australia, Victoria, Australia

[8] Carnegie Applied Rugby Research (CARR) centre, Institute for Sport, Physical Activity and Leisure, Leeds Beckett University, Leeds, UK

[9] Department of Sport Science Innovation, Playermaker, London, United Kingdom

[10] Newcastle Falcons Rugby Club, Newcastle upon Tyne, UK

[11] Institute of sport, Manchester Metropolitan University, Manchester UK

Corresponding Author:

Tzlil Shushan

Email: Tzlil21092@gmail.com

Table S1 Study quality assessment characteristics

Dimension		Description for our meta-analysis
1	Selection of participants	• Reporting clear sample size characteristics (sex, mean age, level etc.)<br>• Reporting new characteristics if n was changed in reliability and/or convergent validity analyses, or across repeated measures (e.g. SMFT protocol, intensity, season phase)
2	Confounding variables	• Environmental details (indoor/outdoor, temperature if outdoor, time during day or AM/PM)<br>• Diet control prior and across measures (e.g. caffeine, alcohol supplements etc.)
3	Intervention (exposure) measurement	• Reporting time between test-retest, or alternatively, reporting why a specific time range was used.<br>• If test-retest was not matched (day/time in the week), providing details on the scheduling and training loads prior the assessments.<br>• Description of standardisation prior the assessment (e.g., warm up)
4	Blinding outcome assessment	• No blinding, but judged to have no influence on results
5	Incomplete outcome data	• Reliability: mean ± SD, TE/CV including method, ICC including type<br>• Convergent validity: HRex mean (±SD), correlation coefficient (*r*)
6	Selective outcome reporting	• No potentially selective/missing results (selective reporting)<br>• Reliability: reporting confidence levels (CI)<br>• Validity: reporting significance and confidence levels (P value and CI)
	Low: All the segments in each domain were fulfilled	
	Unclear: Only part of the segments in each domain were fulfilled	
	High: None of the segments were fulfilled (domain 1-4), less than two reliability results, potentially missing correlation results or concerns for selective reporting (domain 5&6)	

AM morning hours; *CI* confidence intervals; *HRex* exercise heart rate; *n* sample size; *ICC* intraclass correlation; *PM* afternoon hours; *SD* standard deviation; *TE* typical error
