# Supplementary material for: Submaximal Fitness Test in Team Sports: A Systematic Review and Meta-Analysis of Exercise Heart Rate Measurement Properties
Source: Sports Med Open. 2023 Mar 24;9:21. doi: 10.1186/s40798-023-00564-w (PMC10039193; doi:10.1186/s40798-023-00564-w)
Supplement: Supplementary file 6 — Additional file 6. Meta-analysis forest plots of the weighted points estimates. [file 40798_2023_564_MOESM6_ESM.pdf]

Name: Meta-analysis forest plots of the weighted points estimates

Article Title: Submaximal Fitness Test in Team Sports: A Systematic Review and Meta-Analysis of Exercise Heart Rate Measurement Properties

Journal: Sports Medicine – Open

Authors: Tzlil Shushan[1], Ric Lovell[1,2], Martin Buchheit[3,4,5,6], Tannath J. Scott[7,8], Steve Barrett[9], Dean Norris[1] and Shaun J. McLaren[10,11]

[1] School of Health Sciences, Western Sydney University, Sydney, NSW, Australia

[2] Faculty of Science, Medicine and Health, University of Wollongong, Wollongong, NSW, Australia

[3] HIIT Science, Revelstoke, BC, Canada

[4] French National Institute of Sport (INSEP), Laboratory of Sport, Expertise and Performance (EA 7370), Paris, France

[5] Kitman Labs, Performance Research Intelligence Initiative, Dublin, Ireland

[6] Institute for Health and Sport, Victoria University, Melbourne, VIC, Australia

[7] Netball Australia, Victoria, Australia

[8] Carnegie Applied Rugby Research (CARR) centre, Institute for Sport, Physical Activity and Leisure, Leeds Beckett University, Leeds, UK

[9] Department of Sport Science Innovation, Playermaker, London, United Kingdom

[10] Newcastle Falcons Rugby Club, Newcastle upon Tyne, UK

[11] Institute of sport, Manchester Metropolitan University, Manchester UK

Corresponding Author:

Tzlil Shushan

Email: Tzlil21092@gmail.com

Fig. S1

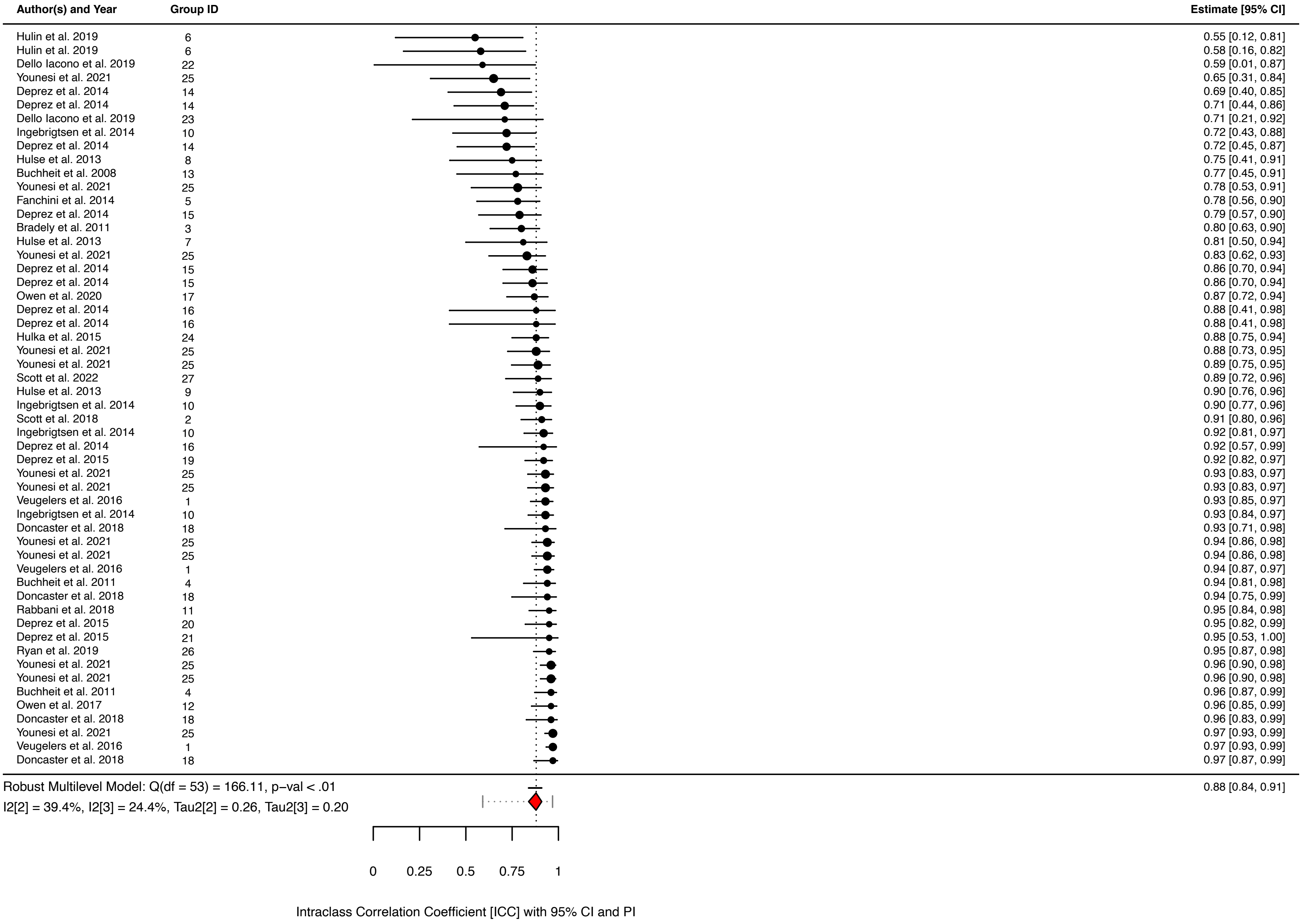
Author(s) and Year
Group ID
Estimate [95% CI]
Hulin et al. 2019 6 0.55 [0.12, 0.81]
Hulin et al. 2019 6 0.58 [0.16, 0.82]
Dello Iacono et al. 2019 22 0.59 [0.01, 0.87]
Younesi et al. 2021 25 0.65 [0.31, 0.84]
Deprez et al. 2014 14 0.69 [0.40, 0.85]
Deprez et al. 2014 14 0.71 [0.44, 0.86]
Dello Iacono et al. 2019 23 0.71 [0.21, 0.92]
Ingebrigtsen et al. 2014 10 0.72 [0.43, 0.88]
Deprez et al. 2014 14 0.72 [0.45, 0.87]
Hulse et al. 2013 8 0.75 [0.41, 0.91]
Buchheit et al. 2008 13 0.77 [0.45, 0.91]
Younesi et al. 2021 25 0.78 [0.53, 0.91]
Fanchini et al. 2014 5 0.78 [0.56, 0.90]
Deprez et al. 2014 15 0.79 [0.57, 0.90]
Bradely et al. 2011 3 0.80 [0.63, 0.90]
Hulse et al. 2013 7 0.81 [0.50, 0.94]
Younesi et al. 2021 25 0.83 [0.62, 0.93]
Deprez et al. 2014 15 0.86 [0.70, 0.94]
Deprez et al. 2014 15 0.86 [0.70, 0.94]
Owen et al. 2020 17 0.87 [0.72, 0.94]
Deprez et al. 2014 16 0.88 [0.41, 0.98]
Deprez et al. 2014 16 0.88 [0.41, 0.98]
Hulka et al. 2015 24 0.88 [0.75, 0.94]
Younesi et al. 2021 25 0.88 [0.73, 0.95]
Younesi et al. 2021 25 0.89 [0.75, 0.95]
Scott et al. 2022 27 0.89 [0.72, 0.96]
Hulse et al. 2013 9 0.90 [0.76, 0.96]
Ingebrigtsen et al. 2014 10 0.90 [0.77, 0.96]
Scott et al. 2018 2 0.91 [0.80, 0.96]
Ingebrigtsen et al. 2014 10 0.92 [0.81, 0.97]
Deprez et al. 2014 16 0.92 [0.57, 0.99]
Deprez et al. 2015 19 0.92 [0.82, 0.97]
Younesi et al. 2021 25 0.93 [0.83, 0.97]
Younesi et al. 2021 25 0.93 [0.83, 0.97]
Veugelers et al. 2016 1 0.93 [0.85, 0.97]
Ingebrigtsen et al. 2014 10 0.93 [0.84, 0.97]
Doncaster et al. 2018 18 0.93 [0.71, 0.98]
Younesi et al. 2021 25 0.94 [0.86, 0.98]
Younesi et al. 2021 25 0.94 [0.86, 0.98]
Veugelers et al. 2016 1 0.94 [0.87, 0.97]
Buchheit et al. 2011 4 0.94 [0.81, 0.98]
Doncaster et al. 2018 18 0.94 [0.75, 0.99]
Rabbani et al. 2018 11 0.95 [0.84, 0.98]
Deprez et al. 2015 20 0.95 [0.82, 0.99]
Deprez et al. 2015 21 0.95 [0.53, 1.00]
Ryan et al. 2019 26 0.95 [0.87, 0.98]
Younesi et al. 2021 25 0.96 [0.90, 0.98]
Younesi et al. 2021 25 0.96 [0.90, 0.98]
Buchheit et al. 2011 4 0.96 [0.87, 0.99]
Owen et al. 2017 12 0.96 [0.85, 0.99]
Doncaster et al. 2018 18 0.96 [0.83, 0.99]
Younesi et al. 2021 25 0.97 [0.93, 0.99]
Veugelers et al. 2016 1 0.97 [0.93, 0.99]
Doncaster et al. 2018 18 0.97 [0.87, 0.99]
Robust Multilevel Model: Q(df = 53) = 166.11, p−val < .01 0.88 [0.84, 0.91]
I2[2] = 39.4%, I2[3] = 24.4%, Tau2[2] = 0.26, Tau2[3] = 0.20
0
0.25
0.5
0.75
1
Intraclass Correlation Coefficient [ICC] with 95% CI and PI

Fig. S2

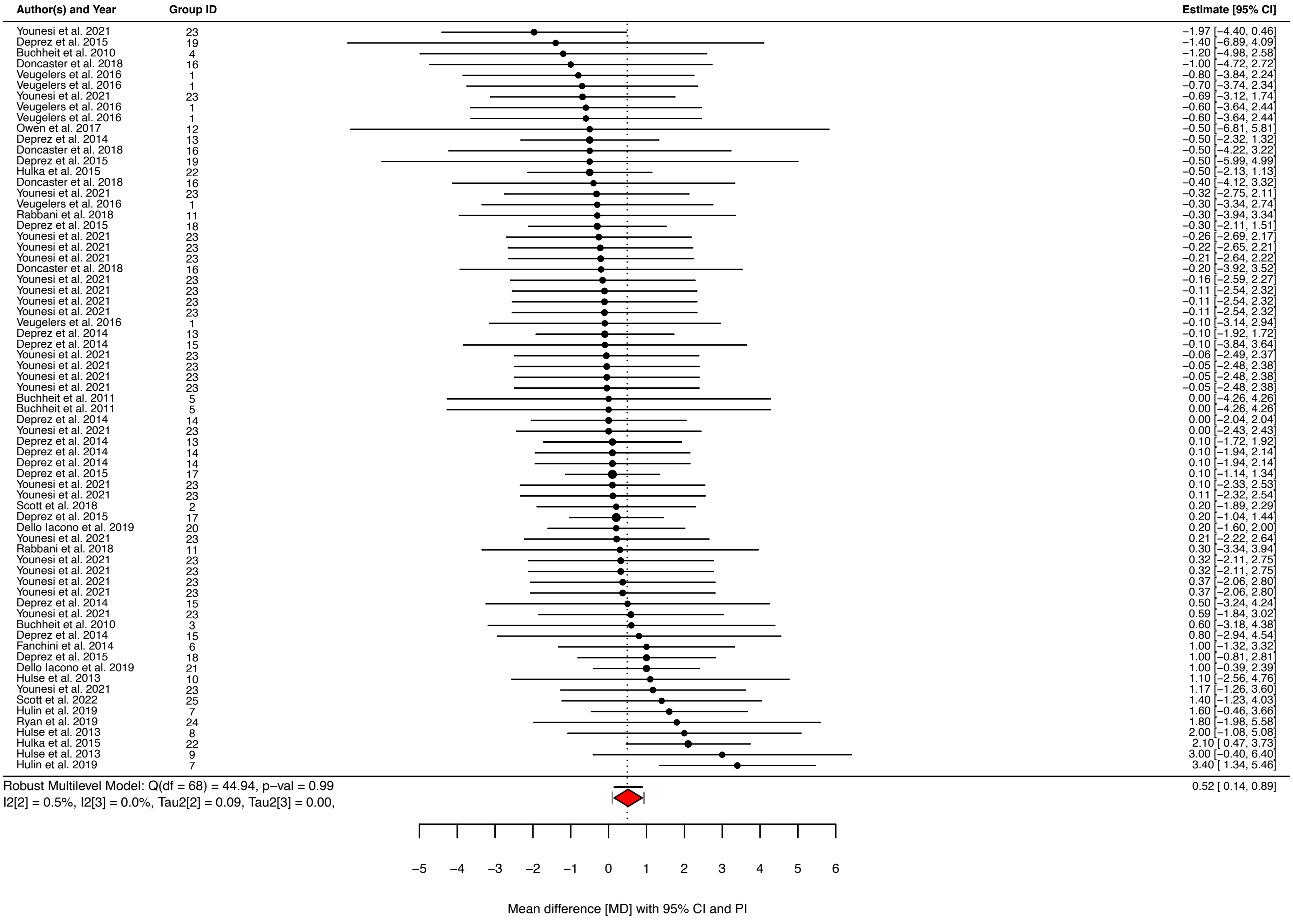
Author(s) and Year Group ID Estimate [95% CI]
Younesi et al. 2021 23 −1.97 [−4.40, 0.46]
Deprez et al. 2015 19 −1.40 [−6.89, 4.09]
Buchheit et al. 2010 4 −1.20 [−4.98, 2.58]
Doncaster et al. 2018 16 −1.00 [−4.72, 2.72]
Veugelers et al. 2016 1 −0.80 [−3.84, 2.24]
Veugelers et al. 2016 1 −0.70 [−3.74, 2.34]
Younesi et al. 2021 23 −0.69 [−3.12, 1.74]
Veugelers et al. 2016 1 −0.60 [−3.64, 2.44]
Veugelers et al. 2016 1 −0.60 [−3.64, 2.44]
Owen et al. 2017 12 −0.50 [−6.81, 5.81]
Deprez et al. 2014 13 −0.50 [−2.32, 1.32]
Doncaster et al. 2018 16 −0.50 [−4.22, 3.22]
Deprez et al. 2015 19 −0.50 [−5.99, 4.99]
Hulka et al. 2015 22 −0.50 [−2.13, 1.13]
Doncaster et al. 2018 16 −0.40 [−4.12, 3.32]
Younesi et al. 2021 23 −0.32 [−2.75, 2.11]
Veugelers et al. 2016 1 −0.30 [−3.34, 2.74]
Rabbani et al. 2018 11 −0.30 [−3.94, 3.34]
Deprez et al. 2015 18 −0.30 [−2.11, 1.51]
Younesi et al. 2021 23 −0.26 [−2.69, 2.17]
Younesi et al. 2021 23 −0.22 [−2.65, 2.21]
Younesi et al. 2021 23 −0.21 [−2.64, 2.22]
Doncaster et al. 2018 16 −0.20 [−3.92, 3.52]
Younesi et al. 2021 23 −0.16 [−2.59, 2.27]
Younesi et al. 2021 23 −0.11 [−2.54, 2.32]
Younesi et al. 2021 23 −0.11 [−2.54, 2.32]
Younesi et al. 2021 23 −0.11 [−2.54, 2.32]
Veugelers et al. 2016 1 −0.10 [−3.14, 2.94]
Deprez et al. 2014 13 −0.10 [−1.92, 1.72]
Deprez et al. 2014 15 −0.10 [−3.84, 3.64]
Younesi et al. 2021 23 −0.06 [−2.49, 2.37]
Younesi et al. 2021 23 −0.05 [−2.48, 2.38]
Younesi et al. 2021 23 −0.05 [−2.48, 2.38]
Younesi et al. 2021 23 −0.05 [−2.48, 2.38]
Buchheit et al. 2011 5 0.00 [−4.26, 4.26]
Buchheit et al. 2011 5 0.00 [−4.26, 4.26]
Deprez et al. 2014 14 0.00 [−2.04, 2.04]
Younesi et al. 2021 23 0.00 [−2.43, 2.43]
Deprez et al. 2014 13 0.10 [−1.72, 1.92]
Deprez et al. 2014 14 0.10 [−1.94, 2.14]
Deprez et al. 2014 14 0.10 [−1.94, 2.14]
Deprez et al. 2015 17 0.10 [−1.14, 1.34]
Younesi et al. 2021 23 0.10 [−2.33, 2.53]
Younesi et al. 2021 23 0.11 [−2.32, 2.54]
Scott et al. 2018 2 0.20 [−1.89, 2.29]
Deprez et al. 2015 17 0.20 [−1.04, 1.44]
Dello Iacono et al. 2019 20 0.20 [−1.60, 2.00]
Younesi et al. 2021 23 0.21 [−2.22, 2.64]
Rabbani et al. 2018 11 0.30 [−3.34, 3.94]
Younesi et al. 2021 23 0.32 [−2.11, 2.75]
Younesi et al. 2021 23 0.32 [−2.11, 2.75]
Younesi et al. 2021 23 0.37 [−2.06, 2.80]
Younesi et al. 2021 23 0.37 [−2.06, 2.80]
Deprez et al. 2014 15 0.50 [−3.24, 4.24]
Younesi et al. 2021 23 0.59 [−1.84, 3.02]
Buchheit et al. 2010 3 0.60 [−3.18, 4.38]
Deprez et al. 2014 15 0.80 [−2.94, 4.54]
Fanchini et al. 2014 6 1.00 [−1.32, 3.32]
Deprez et al. 2015 18 1.00 [−0.81, 2.81]
Dello Iacono et al. 2019 21 1.00 [−0.39, 2.39]
Hulse et al. 2013 10 1.10 [−2.56, 4.76]
Younesi et al. 2021 23 1.17 [−1.26, 3.60]
Scott et al. 2022 25 1.40 [−1.23, 4.03]
Hulin et al. 2019 7 1.60 [−0.46, 3.66]
Ryan et al. 2019 24 1.80 [−1.98, 5.58]
Hulse et al. 2013 8 2.00 [−1.08, 5.08]
Hulka et al. 2015 22 2.10 [0.47, 3.73]
Hulse et al. 2013 9 3.00 [−0.40, 6.40]
Hulin et al. 2019 7 3.40 [1.34, 5.46]
Robust Multilevel Model: Q(df = 68) = 44.94, p−val = 0.99 0.52 [0.14, 0.89]
I2[2] = 0.5%, I2[3] = 0.0%, Tau2[2] = 0.09, Tau2[3] = 0.00,
−5 −4 −3 −2 −1 0 1 2 3 4 5 6
Mean difference [MD] with 95% CI and PI

Fig. S3

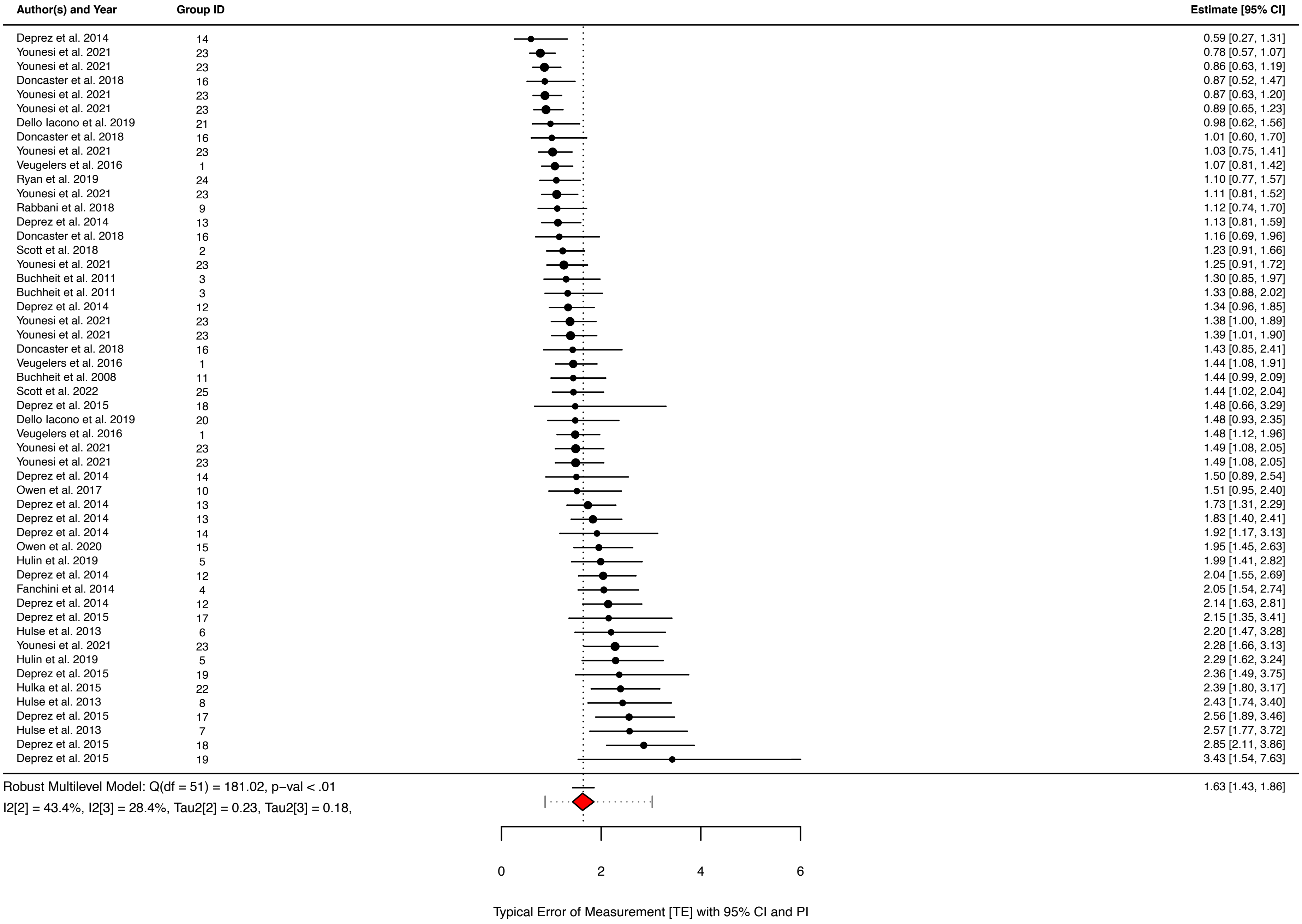
Author(s) and Year
Group ID
Estimate [95% CI]
Deprez et al. 2014 14 0.59 [0.27, 1.31]
Younesi et al. 2021 23 0.78 [0.57, 1.07]
Younesi et al. 2021 23 0.86 [0.63, 1.19]
Doncaster et al. 2018 16 0.87 [0.52, 1.47]
Younesi et al. 2021 23 0.87 [0.63, 1.20]
Younesi et al. 2021 23 0.89 [0.65, 1.23]
Dello Iacono et al. 2019 21 0.98 [0.62, 1.56]
Doncaster et al. 2018 16 1.01 [0.60, 1.70]
Younesi et al. 2021 23 1.03 [0.75, 1.41]
Veugelers et al. 2016 1 1.07 [0.81, 1.42]
Ryan et al. 2019 24 1.10 [0.77, 1.57]
Younesi et al. 2021 23 1.11 [0.81, 1.52]
Rabbani et al. 2018 9 1.12 [0.74, 1.70]
Deprez et al. 2014 13 1.13 [0.81, 1.59]
Doncaster et al. 2018 16 1.16 [0.69, 1.96]
Scott et al. 2018 2 1.23 [0.91, 1.66]
Younesi et al. 2021 23 1.25 [0.91, 1.72]
Buchheit et al. 2011 3 1.30 [0.85, 1.97]
Buchheit et al. 2011 3 1.33 [0.88, 2.02]
Deprez et al. 2014 12 1.34 [0.96, 1.85]
Younesi et al. 2021 23 1.38 [1.00, 1.89]
Younesi et al. 2021 23 1.39 [1.01, 1.90]
Doncaster et al. 2018 16 1.43 [0.85, 2.41]
Veugelers et al. 2016 1 1.44 [1.08, 1.91]
Buchheit et al. 2008 11 1.44 [0.99, 2.09]
Scott et al. 2022 25 1.44 [1.02, 2.04]
Deprez et al. 2015 18 1.48 [0.66, 3.29]
Dello Iacono et al. 2019 20 1.48 [0.93, 2.35]
Veugelers et al. 2016 1 1.48 [1.12, 1.96]
Younesi et al. 2021 23 1.49 [1.08, 2.05]
Younesi et al. 2021 23 1.49 [1.08, 2.05]
Deprez et al. 2014 14 1.50 [0.89, 2.54]
Owen et al. 2017 10 1.51 [0.95, 2.40]
Deprez et al. 2014 13 1.73 [1.31, 2.29]
Deprez et al. 2014 13 1.83 [1.40, 2.41]
Deprez et al. 2014 14 1.92 [1.17, 3.13]
Owen et al. 2020 15 1.95 [1.45, 2.63]
Hulin et al. 2019 5 1.99 [1.41, 2.82]
Deprez et al. 2014 12 2.04 [1.55, 2.69]
Fanchini et al. 2014 4 2.05 [1.54, 2.74]
Deprez et al. 2014 12 2.14 [1.63, 2.81]
Deprez et al. 2015 17 2.15 [1.35, 3.41]
Hulse et al. 2013 6 2.20 [1.47, 3.28]
Younesi et al. 2021 23 2.28 [1.66, 3.13]
Hulin et al. 2019 5 2.29 [1.62, 3.24]
Deprez et al. 2015 19 2.36 [1.49, 3.75]
Hulka et al. 2015 22 2.39 [1.80, 3.17]
Hulse et al. 2013 8 2.43 [1.74, 3.40]
Deprez et al. 2015 17 2.56 [1.89, 3.46]
Hulse et al. 2013 7 2.57 [1.77, 3.72]
Deprez et al. 2015 18 2.85 [2.11, 3.86]
Deprez et al. 2015 19 3.43 [1.54, 7.63]
Robust Multilevel Model: Q(df = 51) = 181.02, p−val < .01 1.63 [1.43, 1.86]
I2[2] = 43.4%, I2[3] = 28.4%, Tau2[2] = 0.23, Tau2[3] = 0.18,
0
2
4
6
Typical Error of Measurement [TE] with 95% CI and PI

Fig. S4

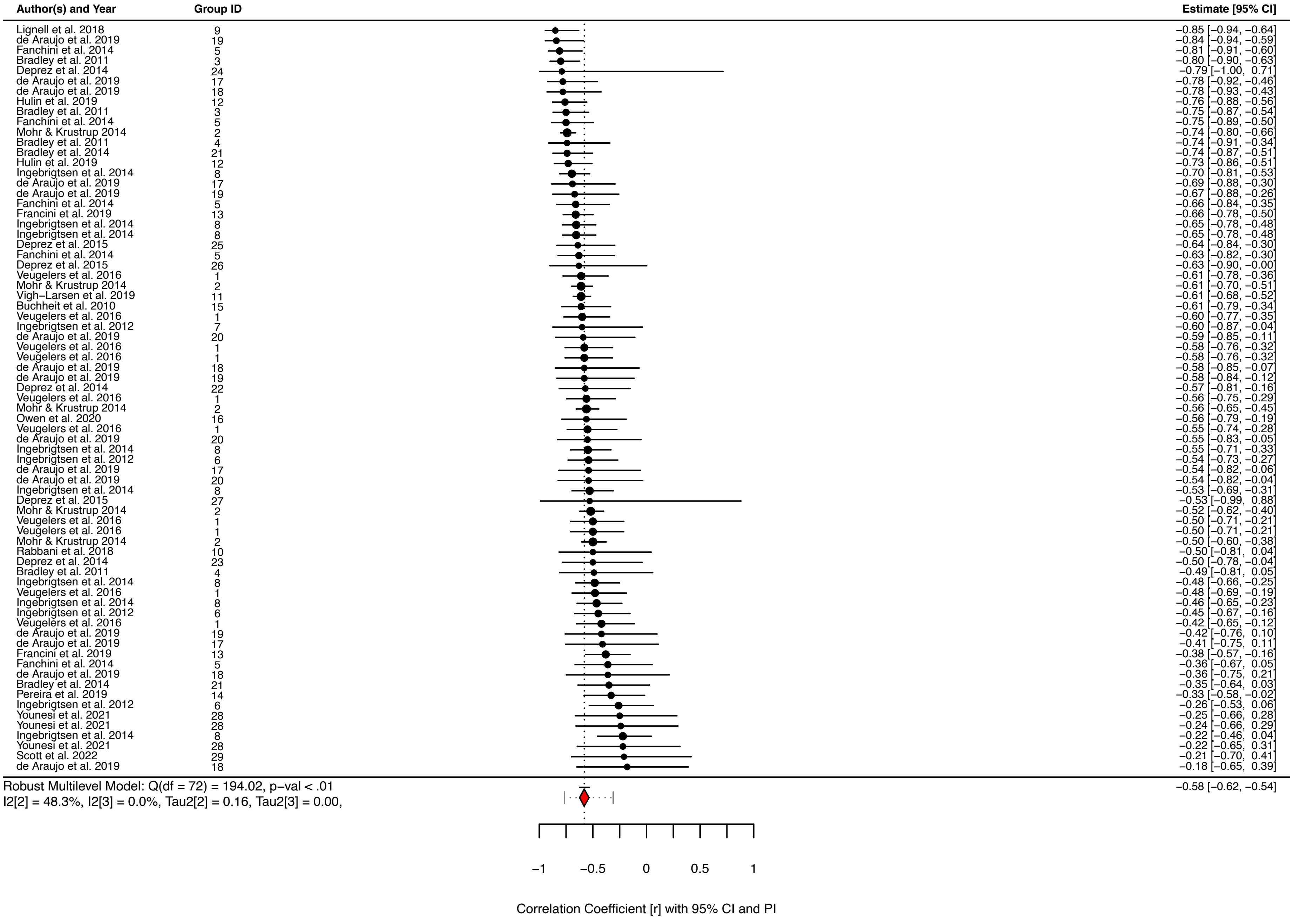
Author(s) and Year
Group ID
Estimate [95% CI]
Lignell et al. 2018 9 −0.85 [−0.94, −0.64]
de Araujo et al. 2019 19 −0.84 [−0.94, −0.59]
Fanchini et al. 2014 5 −0.81 [−0.91, −0.60]
Bradley et al. 2011 3 −0.80 [−0.90, −0.63]
Deprez et al. 2014 24 −0.79 [−1.00, 0.71]
de Araujo et al. 2019 17 −0.78 [−0.92, −0.46]
de Araujo et al. 2019 18 −0.78 [−0.93, −0.43]
Hulin et al. 2019 12 −0.76 [−0.88, −0.56]
Bradley et al. 2011 3 −0.75 [−0.87, −0.54]
Fanchini et al. 2014 5 −0.75 [−0.89, −0.50]
Mohr & Krustrup 2014 2 −0.74 [−0.80, −0.66]
Bradley et al. 2011 4 −0.74 [−0.91, −0.34]
Bradley et al. 2014 21 −0.74 [−0.87, −0.51]
Hulin et al. 2019 12 −0.73 [−0.86, −0.51]
Ingebrigtsen et al. 2014 8 −0.70 [−0.81, −0.53]
de Araujo et al. 2019 17 −0.69 [−0.88, −0.30]
de Araujo et al. 2019 19 −0.67 [−0.88, −0.26]
Fanchini et al. 2014 5 −0.66 [−0.84, −0.35]
Francini et al. 2019 13 −0.66 [−0.78, −0.50]
Ingebrigtsen et al. 2014 8 −0.65 [−0.78, −0.48]
Ingebrigtsen et al. 2014 8 −0.65 [−0.78, −0.48]
Deprez et al. 2015 25 −0.64 [−0.84, −0.30]
Fanchini et al. 2014 5 −0.63 [−0.82, −0.30]
Deprez et al. 2015 26 −0.63 [−0.90, −0.00]
Veugelers et al. 2016 1 −0.61 [−0.78, −0.36]
Mohr & Krustrup 2014 2 −0.61 [−0.70, −0.51]
Vigh−Larsen et al. 2019 11 −0.61 [−0.68, −0.52]
Buchheit et al. 2010 15 −0.61 [−0.79, −0.34]
Veugelers et al. 2016 1 −0.60 [−0.77, −0.35]
Ingebrigtsen et al. 2012 7 −0.60 [−0.87, −0.04]
de Araujo et al. 2019 20 −0.59 [−0.85, −0.11]
Veugelers et al. 2016 1 −0.58 [−0.76, −0.32]
Veugelers et al. 2016 1 −0.58 [−0.76, −0.32]
de Araujo et al. 2019 18 −0.58 [−0.85, −0.07]
de Araujo et al. 2019 19 −0.58 [−0.84, −0.12]
Deprez et al. 2014 22 −0.57 [−0.81, −0.16]
Veugelers et al. 2016 1 −0.56 [−0.75, −0.29]
Mohr & Krustrup 2014 2 −0.56 [−0.65, −0.45]
Owen et al. 2020 16 −0.56 [−0.79, −0.19]
Veugelers et al. 2016 1 −0.55 [−0.74, −0.28]
de Araujo et al. 2019 20 −0.55 [−0.83, −0.05]
Ingebrigtsen et al. 2014 8 −0.55 [−0.71, −0.33]
Ingebrigtsen et al. 2012 6 −0.54 [−0.73, −0.27]
de Araujo et al. 2019 17 −0.54 [−0.82, −0.06]
de Araujo et al. 2019 20 −0.54 [−0.82, −0.04]
Ingebrigtsen et al. 2014 8 −0.53 [−0.69, −0.31]
Deprez et al. 2015 27 −0.53 [−0.99, 0.88]
Mohr & Krustrup 2014 2 −0.52 [−0.62, −0.40]
Veugelers et al. 2016 1 −0.50 [−0.71, −0.21]
Veugelers et al. 2016 1 −0.50 [−0.71, −0.21]
Mohr & Krustrup 2014 2 −0.50 [−0.60, −0.38]
Rabbani et al. 2018 10 −0.50 [−0.81, 0.04]
Deprez et al. 2014 23 −0.50 [−0.78, −0.04]
Bradley et al. 2011 4 −0.49 [−0.81, 0.05]
Ingebrigtsen et al. 2014 8 −0.48 [−0.66, −0.25]
Veugelers et al. 2016 1 −0.48 [−0.69, −0.19]
Ingebrigtsen et al. 2014 8 −0.46 [−0.65, −0.23]
Ingebrigtsen et al. 2012 6 −0.45 [−0.67, −0.16]
Veugelers et al. 2016 1 −0.42 [−0.65, −0.12]
de Araujo et al. 2019 19 −0.42 [−0.76, 0.10]
de Araujo et al. 2019 17 −0.41 [−0.75, 0.11]
Francini et al. 2019 13 −0.38 [−0.57, −0.16]
Fanchini et al. 2014 5 −0.36 [−0.67, 0.05]
de Araujo et al. 2019 18 −0.36 [−0.75, 0.21]
Bradley et al. 2014 21 −0.35 [−0.64, 0.03]
Pereira et al. 2019 14 −0.33 [−0.58, −0.02]
Ingebrigtsen et al. 2012 6 −0.26 [−0.53, 0.06]
Younesi et al. 2021 28 −0.25 [−0.66, 0.28]
Younesi et al. 2021 28 −0.24 [−0.66, 0.29]
Ingebrigtsen et al. 2014 8 −0.22 [−0.46, 0.04]
Younesi et al. 2021 28 −0.22 [−0.65, 0.31]
Scott et al. 2022 29 −0.21 [−0.70, 0.41]
de Araujo et al. 2019 18 −0.18 [−0.65, 0.39]
Robust Multilevel Model: Q(df = 72) = 194.02, p−val < .01
I2[2] = 48.3%, I2[3] = 0.0%, Tau2[2] = 0.16, Tau2[3] = 0.00,
−0.58 [−0.62, −0.54]
−1
−0.5
0
0.5
1
Correlation Coefficient [r] with 95% CI and PI
