# Supplementary material for: Submaximal Fitness Test in Team Sports: A Systematic Review and Meta-Analysis of Exercise Heart Rate Measurement Properties
Source: Sports Med Open. 2023 Mar 24;9:21. doi: 10.1186/s40798-023-00564-w (PMC10039193; doi:10.1186/s40798-023-00564-w)
Supplement: Supplementary file 7 — Additional file 7. Sensitivity and influence analyses from metaanalysis of measurement properties. [file 40798_2023_564_MOESM7_ESM.pdf]

Name: Sensitivity and influence analyses from meta-analysis of measurement properties

Article Title: Submaximal Fitness Test in Team Sports: A Systematic Review and Meta-Analysis of Exercise Heart Rate Measurement Properties

Journal: Sports Medicine – Open

Authors: Tzlil Shushan[1], Ric Lovell[1,2], Martin Buchheit[3,4,5,6], Tannath J. Scott[7,8], Steve Barrett[9], Dean Norris[1] and Shaun J. McLaren[10,11]

[1] School of Health Sciences, Western Sydney University, Sydney, NSW, Australia

[2] Faculty of Science, Medicine and Health, University of Wollongong, Wollongong, NSW, Australia

[3] HIIT Science, Revelstoke, BC, Canada

[4] French National Institute of Sport (INSEP), Laboratory of Sport, Expertise and Performance (EA 7370), Paris, France

[5] Kitman Labs, Performance Research Intelligence Initiative, Dublin, Ireland

[6] Institute for Health and Sport, Victoria University, Melbourne, VIC, Australia

[7] Netball Australia, Victoria, Australia

[8] Carnegie Applied Rugby Research (CARR) centre, Institute for Sport, Physical Activity and Leisure, Leeds Beckett University, Leeds, UK

[9] Department of Sport Science Innovation, Playermaker, London, United Kingdom

[10] Newcastle Falcons Rugby Club, Newcastle upon Tyne, UK

[11] Institute of sport, Manchester Metropolitan University, Manchester UK

Corresponding Author:

Tzlil Shushan

Email: Tzlil21092@gmail.com

Figure S1–4 represent Baujat and Cook's distance plots for influence analysis and potential outliers: **(1)** Intraclass Correlation Coefficient (ICC); **(2)**: Mean Difference (MD); **(3)**: Typical Error (TE); and **(4)**: Correlation Coefficient (*r*). The horizontal red line represents the value of three times cook's distance mean. The exclusion of potential outliers in all datasets did not have a practically meaningful influence on the results obtained in the original models – ICC: 0.88 (95%CI: 0.84 to 0.92, 95%PI: 0.60 to 0.97), MD: 0.34 (95%CI: 0.02 to 0.7, 95%PI: 0.02 to 0.67), TE: 1.57 (95%CI: 1.37 to 1.8, 95%PI: 0.88 to 2.80), correlation coefficient (*r*): –0.58 (95%CI: –0.62 to –0.54, 95%PI: –0.73 to –0.37).

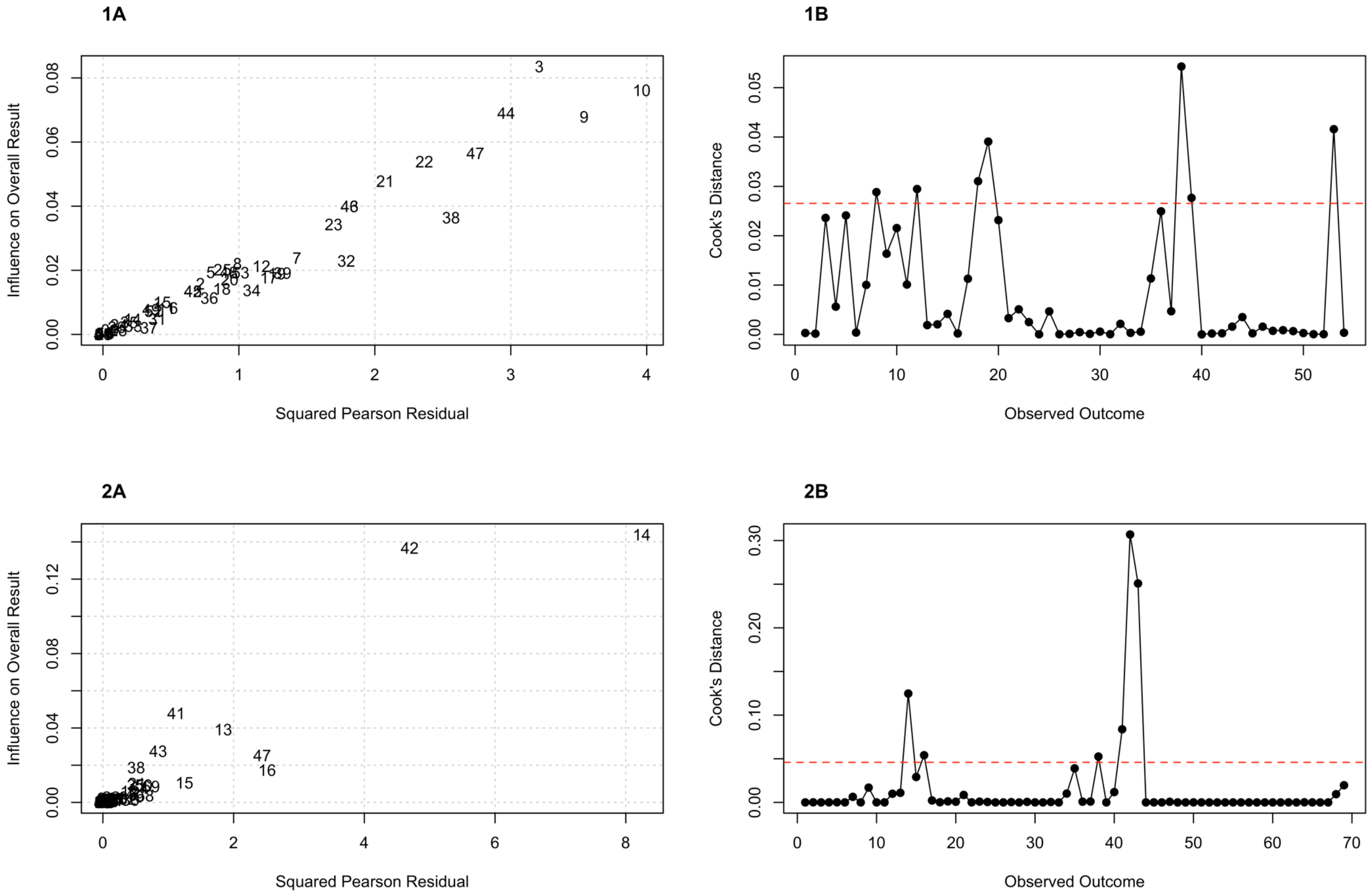
1A
Influence on Overall Result
Squared Pearson Residual
1B
Cook's Distance
Observed Outcome
2A
Influence on Overall Result
Squared Pearson Residual
2B
Cook's Distance
Observed Outcome

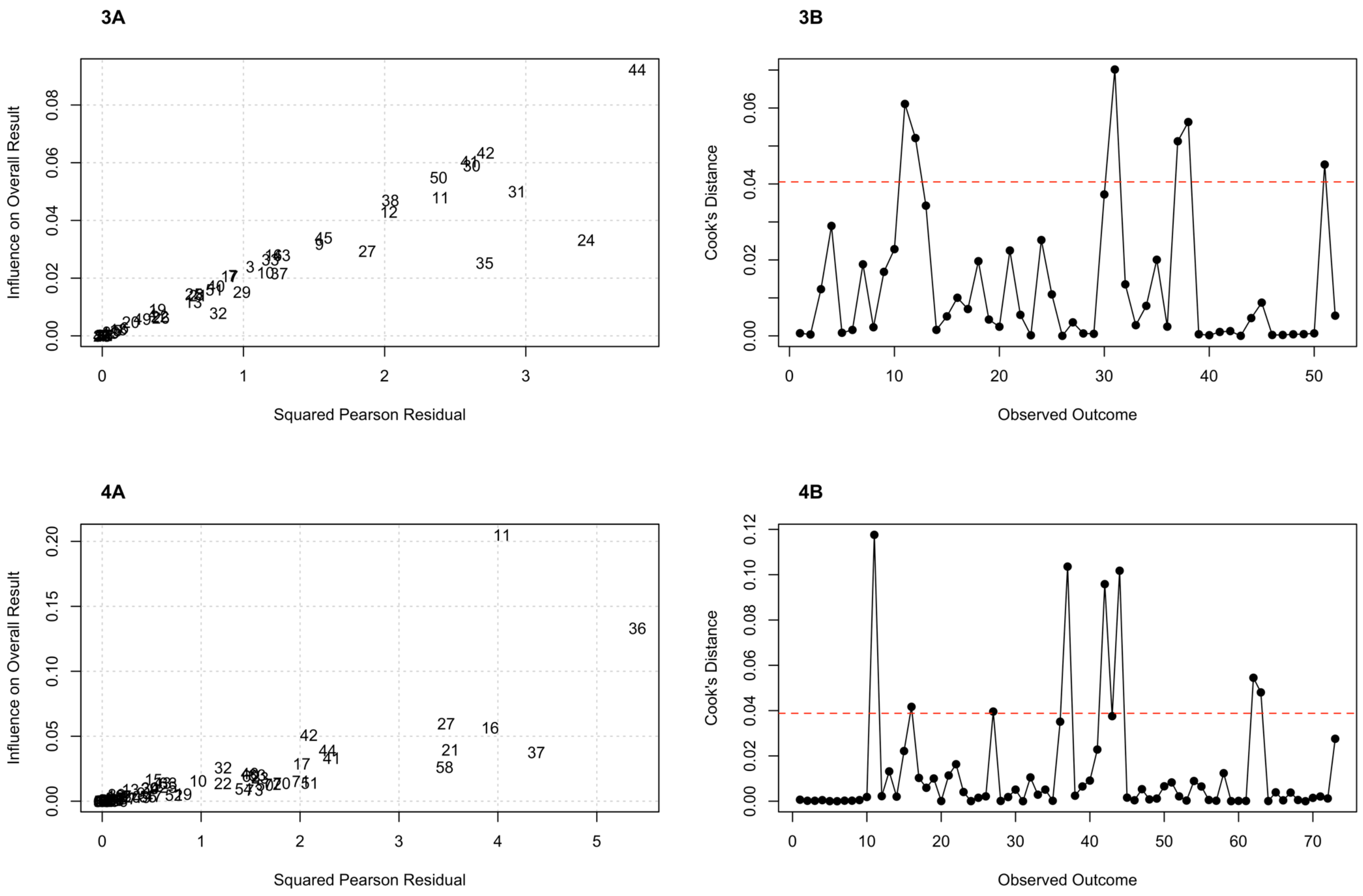
3A
Influence on Overall Result
Squared Pearson Residual
3B
Cook's Distance
Observed Outcome
4A
Influence on Overall Result
Squared Pearson Residual
4B
Cook's Distance
Observed Outcome
